# MoS_2_‐Based Nanocomposites for Electrochemical Energy Storage

**DOI:** 10.1002/advs.201600289

**Published:** 2016-12-06

**Authors:** Tianyi Wang, Shuangqiang Chen, Huan Pang, Huaiguo Xue, Yan Yu

**Affiliations:** ^1^ College of Chemistry and Chemical EngineeringYangzhou UniversityYangzhouJiangsu225002China; ^2^ Key Laboratory of Advanced Energy Materials Chemistry (Ministry of Education)Nankai UniversityTianjin300071China; ^3^ Centre for Clean Energy TechnologySchool of Chemistry and Forensic ScienceUniversity of Technology Sydney BroadwaySydneyAustralia; ^4^ Key Laboratory of Materials for Energy ConversionChinese Academy of SciencesDepartment of Materials Science and EngineeringUniversity of Science and Technology of ChinaHefeiAnhui230026China

**Keywords:** energy storage, lithium ion batteries, molybdenum disulfide, sodium ion batteries, supercapacitors

## Abstract

Typical layered transition‐metal chalcogenide materials, in particular layered molybdenum disulfide (MoS_2_) nanocomposites, have attracted increasing attention in recent years due to their excellent chemical and physical properties in various research fieldsHere, a general overview of synthetic MoS_2_ based nanocomposites via different preparation approaches and their applications in energy storage devices (Li‐ion battery, Na‐ion battery, and supercapacitor) is presented. The relationship between morphologies and the electrochemical performances of MoS_2_‐based nanocomposites in the three typical and promising rechargeable systems is also discussed. Finally, perspectives on major challenges and opportunities faced by MoS_2_‐based materials to address the practical problems of MoS_2_‐based materials are presented.

## 1 Introduction

As is known, accompanied with the increasing consumption of fossil fuel and the vast amount of energy demands,^1^ cutting‐edge energy storage technologies with environmentally friendly and low cost features are desired for society in the future and can provide far‐reaching benefits.^2^ In recent years, lithium ion batteries (LIB), lithium sulfur batteries, sodium ion batteries (SIB), and supercapacitors have shown their unique potentials and various advantages, including high energy density, long cycle life, and low self‐discharge features.^3^, ^4^ Though limitations on the capacities and cycling performances of these rechargeable devices still exist, recent research progress with improved performance for those rechargeable systems give us many significant hints to further enhance the electrochemical performance of energy storage devices. Among the developments of rechargeable energy storage devices, including various electrodes, electrolytes, and many types of newly designed rechargeable systems, nanostructured materials with elaborate‐designed morphologies have a place for the following reasons: i) nanomaterials with newly designed architectures, highly flexible feature, and free‐standing or binder‐free traits for long‐term cycling performances;^5^, ^6^, ^7^, ^8^ ii) creating highly reactive sites with post treatments and enhancing catalytic activity;^9^, ^10^, ^11^ iii) increasing conductivity via additional carbon or conductive polymers coating techniques of nanostructured materials to reduce inner electrochemical resistance.^12^, ^13^

Most importantly, the abrupt prosperity of investigations on low‐dimensional nanostructured (LDN) materials have developed a new brunch in material fields.^14^, ^15^, ^16^, ^17^, ^18^ Accompanied with many advanced LDN materials, mono‐ and multilayered transition metal dichalcogenide (TMD) nanosheets have attracted intensive interest and have shown broad and tremendous application prospects, such as photology, electrochemistry, and electricity magnetism.^19^, ^20^, ^21^ The TMD materials include the disulfides, diselenides, and ditellurides of Mo, Ti, Zr, Hf, V, Ta, Nb, Cr, and W, which arrange themselves in various crystalline forms, yet only the Mo and W compounds (disulfides, diselenides, and ditellurides) form a hexagonal‐type crystal structure. Because of their special structure and versatile properties, the LDN nanofibers and nanosheets, which are arranged by LDN materials, can act as satisfactory substitutions for the most popular materials, such as graphene and carbon nanotubes.^22^, ^23^

**Figure 1:**
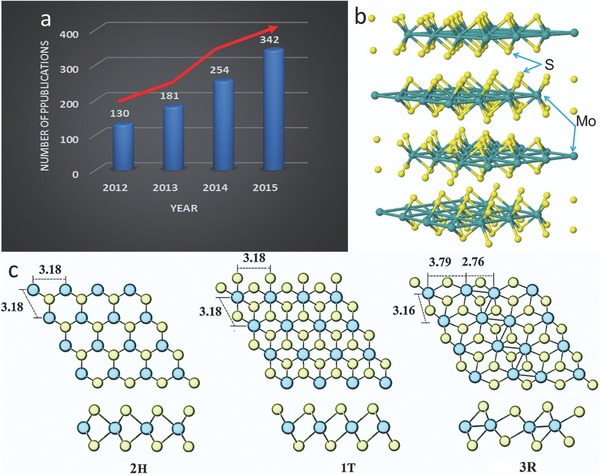
a) Comparison of the number of scientific publications with the search keyword “MoS_2_ composite”, as searched by SciFinder Scholar (March 27, 2016). b) The scheme of MoS_2_ mo lecular structure. c) Three main types of atom arrangements of MoS_2_ (2H‐MoS_2_, 1T‐MoS_2_ and 3R MoS_2_). Reproduced with permission.^31^ Copyright 2014, Royal Society of Chemistry.

Among these transition metals, molybdenum disulfide (MoS_2_) has played a key role in recent few years and it has been investigated in high frequency. **Figure** **1**a shows the outstanding popular investigations of MoS_2_ materials in recent years. This shows visually that the MoS_2_ material is attracting increasing interest in the recent four years, based on the quickly increasing numbers of scientific publications. Until now, wide ranging categories of applications based on MoS_2_ nanomaterials have been investigated in energy storage devices, electronic sensors, and biomedical engineering instruments.^24^, ^25^ Compared with graphene or other monolayered nanomaterials, each piece of a MoS_2_ layer consists of two S layers and one Mo layer (S‐Mo‐S), which is bonded to adjacent MoS_2_ layers by weak van der Waals force and the corresponding molecular structure scheme visualized by JSmol is shown in Figure 1b.^26^, ^27^, ^28^, ^29^, ^30^ This outstanding mechanical property of MoS_2_ makes it an ideal solid lubrication material and it is possible to be used in many specific harsh environments such as space crafts and artificial satellites.

Based on many investigations of the crystal structure of MoS_2_, three main types of atom arrangements (1T‐MoS_2_, 2H‐MoS_2_, and 3R‐MoS_2_) have been found, and the typical structures of MoS_2_ are presented in Figure 1c.^31^ In these three types, 2H and 3R types of MoS_2_ are existent in nature and both of them have trigonal prismatic coordination (the stacking sequences are ABA and ABC, respectively). However, the 3R type MoS_2_, showing three layers per unit in a rhombohedral symmetry, is easily transformed to the more stable 2H type during heating. However, 1T‐MoS_2_ is a type of artificially prepared structure. Compared to 1T‐MoS_2_ (due to its arrangement of the Mo atom, 1T‐MoS_2_ is metastable and metallic as shown in Figure 1c), 2H‐MoS_2_ and 3R‐MoS_2_ have semiconducting properties. According to previous reports, 1T and 2H MoS_2_ can convert to each other by electric doping, annealing, applying strain and electron‐beam irradiating, which has immense technical meaning due to the tunable electronic properties without introducing heteroatoms. The mixture of 1T and 2H MoS_2_ composites have also been widely investigated for sensors and other applications.^32^, ^33^, ^34^

**Figure 2:**
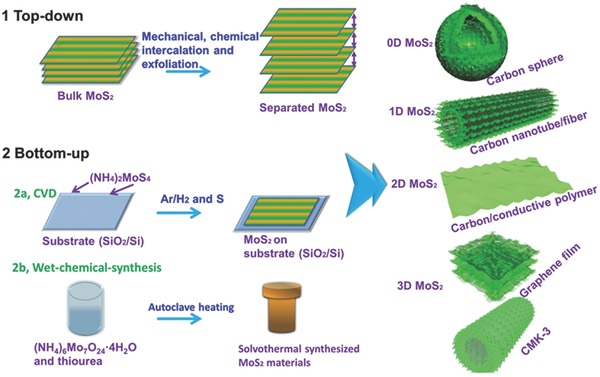
The schematic illustration of the preparation methods of MoS_2_. 1) Top‐down approach using bulk MoS_2_ as precursor. 2) Bottom‐up approach, including CVD method and wet‐chemical‐synthesis method. The as‐prepared MoS_2_ can arrange to different dimensions with different matrices (carbon sphere, carbon nanotube, carbon/conductive polymer coating, graphene film, and CMK‐3). Reproduced with permission.^33^ Copyright 2015, Royal Society of Chemistry.

Until now, the study of the pure MoS_2_ layers has been matured, and research attentions have turned to other special structure of MoS_2,_ which give it exceptional advantages combined with other nanomaterials. The synthesis method of MoS_2_ nanocomposites and their applications in sensors have been discussed in detail by Wei^33^ and Li.^35^ There are two typical methods to prepare MoS_2_: top‐down and bottom‐up. A top‐down method starts from bulk MoS_2_ crystals as the raw material, and the bulk MoS_2_ crystals can be easily exfoliated to atomically thin layers via mechanical cleavage, high‐energy sonication, and liquid exfoliation or chemical intercalation‐exfoliation methods based on the special feature of MoS_2_ (interlayer contacted via the weak van der Waals force).^36^, ^37^, ^38^, ^39^ The bottom‐up method can be divided into two approaches: 1) chemical vapor deposition (two‐step thermolysis using ammonium thiomolybdeates or molybodenum trioxide as the precursor followed by sulfidation with sulfur) method with the features of high quality, controllable thickness, and compatible substrates; and 2) wet‐chemical synthesis including hydrothermal and solvothermal ((NH_4_)_6_Mo_7_O_24_·4H_2_O and thiourea have been utilized as the precursors), showing relative lower crystal quality yet abundant active sites and easy to form morphologies with various substrates (such as hollow carbon spheres, carbon nanotube/nanofibers, amorphous carbon/conductive polymer coatings, graphene films, and CMK‐3). The preparation methods and typical morphologies of MoS_2_ are summarized in **Figure** **2**, which may inspire researchers to prepare the favorable materials with active phases for different applications.

In this article, we summarize new preparation methods forf MoS_2_‐based materials and describe their applications in three types of energy storage devices (lithium ion batteries, sodium ion batteries, and supercapacitors) in detail. We also discuss the relationships between the tuned features and the electrochemical performances of MoS_2_ materials. Finally, we give a perspective on major challenges and opportunities faced by MoS_2_‐based materials to address the practical problems of MoS_2_‐based materials.

## 2 Energy Storage Device Applications

### 2.1 Lithium Ion Batteries

With the rapid developments of mobile electric devices and electric vehicles, the demand for high energy batteries with highly efficient energy storage is more urgent. Relying on its high energy density value (up to 400 Wh Kg^−1^ in theory) and capacity (755 mAh g^−1^), lower volume ratio and higher stability (compared with some traditional batteries), the Li ion battery is regarded as the most promising energy storage system to power millions of portable devices and electric vehicles.^40^, ^41^, ^42^, ^43^ Moreover, it can store the non‐continuous energy harvesting from other alternative clean energies (wind energy, solar energy, and hydroenergy) as chemical energy and release it as electric energy to power various devices when needed.^44^ In this field, Goodenough, Tarascon, J. Dahn, L. Chen, and Zhao et al. have carried out intensive investigations on new‐types of cathodes and have made meaningful discoveries on the lithiation/delithiation mechanisms as well as anodes with high capacities and stable cycling performances.^45^, ^46^, ^47^, ^48^, ^49^, ^50^, ^51^, ^52^ Nowadays, Li‐ion batteries are widely used in portable electric devices and electric vehicles with high energy density, thus reducing the consumption to fossil fuel in some degree.^53^, ^54^ However, many problems continue to restrict the promotion of LIBs.^55^, ^56^ First, the severe volume expansion of the anode material is inevitable, which can cause electrode pulverization and loss of contact with current collectors during long‐term cycles.^57^ Second, overcharge occurs with the possibility of shortcut inside of LIB. Third, the passivating solid electrolyte interface has irreversible effects on the LIB cycling capability in the first few cycles, resulting in low Coulombic efficiency.^58^ To solve the problems mentioned above, researchers are trying to excavate the potentials of LIBs in three main ways: i) by preparing novel materials with a protection layer to control the large volume changes, which has larger chemical potential differences to enhance the energy density; ii) by additional of protection circuits to avoid overcharge, and by storing LIBs in a cool place with at least 40% charge to resist the aging effect; and iii) by designing a special material architecture to resist the further growth of solid electrolyte interfaces.

The properties and electrochemical performances of MoS_2_ composites were reviewed by Mitlin et al., confirming the theoretical capacity and the intercalation and conversion reactions of MoS_2_.^178^ Compared to other anode materials for LIB, MoS_2_ with a layered structure exhibits a high rechargeable capacity of 1290 mAh g^−1^ and stable cycling performance, which is regarded as one of the most promising materials for LIBs and has attracted much attention in recent years.^58^, ^59^, ^60^, ^61^, ^62^, ^63^, ^64^, ^65^, ^66^, ^67^, ^182^, ^183^ It was discovered that MoS_2_ has advantages for rate capacity due to the layered structure and good capacity retention ratio because of the high reversibility during redox reactions with lithium ions. Based on the literature and on experimental results,^178^, ^179^, ^180^, ^181^, ^182^, ^183^ the lithiation/delithiation mechanism of MoS_2_ is mainly divided into two sections (3–1.1 V and 1.1–0.01 V). The intercalation of MoS_2_ with lithium ions occurs between 3 V and 1.1 V during the discharge process, which shown in the redox reaction equation^58^:

(1) $${\mathrm{MoS}}_{2}+x\mathrm{Li}+xe^{-}⇋{\mathrm{Li}}_{x}{\mathrm{MoS}}_{2}(0\leq x\leq 1)$$

The lithium ion insertion is fully reversible if the MoS_2_ electrode is cycled between 3 V and 1.1 V with an intercalation reaction mechanism, contributing a capacity of 167 mAh g^−1^. Further discharge process lead to the decomposition of Li*_x_*MoS_2_ and the formation lithium sulfide as the final product, which might follow the conversion reactions:

(2) $$({\mathrm{Li}}_{x}{\mathrm{MoS}}_{2}+(4-x){\mathrm{Li}}^{+}+4e^{-}⇋\mathrm{Mo}+2{\mathrm{Li}}_{2}S$$

(3) $$S+2{\mathrm{Li}}^{+}+2e⇋{\mathrm{Li}}_{2}S$$

Even though, many reported results support the conversion reactions of Li*_x_*MoS_2_ after 1.1 V, contributing a capacity of 669 mAh g^−1^, there is no solid evidence to support Equation (3), and it is almost impossible to re‐form MoS_2_ at high rates due to the formation of metallic molybdenum. It is believed that the additional capacity comes from the nanostructured carbon materials of MoS_2_ composites, and cycling performance of MoS_2_ can also be enhanced by minimizing particle size and assembling with various carbon‐based materials. This discharge product of Li_2_S might cause other redox reactions to generate S as the charge product during recharge, which makes it face the shuttle effect of lithium sulfur batteries and arouse irreversible capacity loss.^58^ However, many previous reports demonstrate highly reversible cyclability with different structural modification strategies to restrain the shuttle effect and volume expansion. Generally speaking, MoS_2_ composite materials can be divided to three categories based on the dimension of the matrix: low dimensional (MoS_2_/carbon spheres, MoS_2_/carbon nanotubes/nanofibers), two dimensional (monolayered MoS_2_ nanosheets), and three dimensional (the free‐standing MoS_2_/graphene film, mesoporous carbon/MoS_2_ composites and other MoS_2_ materials with special structures).^61^, ^62^, ^68^, ^69^, ^70^

#### 2.1.1 Low Dimensional MoS_2_

**Figure 3:**
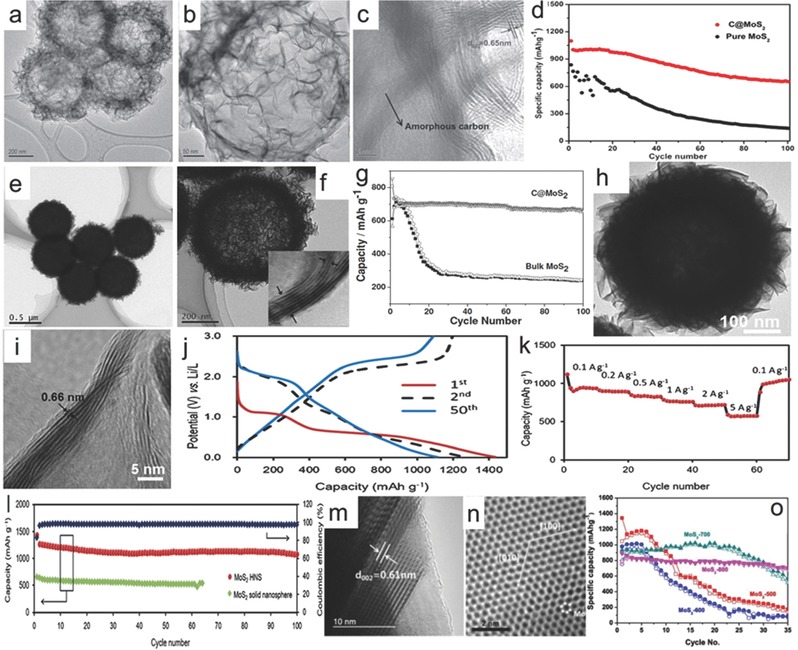
a–c) Low and high magnification TEM images of C@MoS_2_ spheres. d) Cycling performance of MoS_2_ and C@MoS_2_ electrodes at the current density of 0.2 A g^−1^. Reproduced with permission.^62^ Copyright 2016, IOP Publishing Ltd. e,f) Low and high magnification TEM images of ultrathin MoS_2_ nanosheets taken at the edge of C@MoS_2_ microsphere. g) Long‐term cycling performances of C@MoS_2_ and bulk MoS_2_ electrodes. Reproduced with permission.^70^ h‐i) FESEM image, low and high magnification TEM images of MoS_2_ HNS after annealing in H_2_/Ar at 700 for 2h. j) Charge/discharge voltage profiles at 0.5 A g^−1^. k) Rate capability test. l) Cycling performance at 0.5 A g^−1^ of the MoS_2_ HNS and of MoS_2_. Reproduced with permission.^74^ m,n) Low and high resolution TEM images of MoS_2_‐800 nanoparticles. o) Cycling performances of MoS_2_ nanoparticles at different temperatures. Reproduced with permission.^76^ Copyright 2016 Royal Society of Chemistry.

With the assistant of carbon spheres, the MoS_2_ layer can be arranged to zero dimensional spheres to decrease the mechanical strain generated during the conversion reaction with lithium ions and to convert the inner strain due to robust carbon spheres, which is beneficial to the long‐term cycling performance. The 0D MoS_2_/C spheres have been demonstrated as good candidates for LIBs that show quite impressive performances.^62^, ^70^ For example, Dong and co‐workers prepared the hollow MoS_2_/carbon spheres (MoS_2_/C) using a simple hydrothermal method with the hollow carbon spheres as the matrix and SiO_2_ nanospheres as the sacrificed template. The thin MoS_2_ nanolayers with a layer distance of 0.65 nm are homogenously wrapped on the surface of hollow carbon nanospheres and show a monodispersed state, which can be seen in **Figure** **3**a–c. The MoS_2_/C electrode retained a high reversible capacity of 750 mAh g^−1^ after 100 cycles, which was much higher than that of the bare MoS_2_ electrode (around 140 mAh g^−1^) as shown in Figure 3d. That is ascribed to the large free spaces of hollow carbon spheres to suppress the aggregation of MoS_2_ and enhance the surface conductivity and charge transfer.^62^ A similar strategy using hollow carbon spheres and a carbon sheath was applied by Lou et al. and Gao et al., demonstrating the improved electrochemical performance (around 800 mAh g^−1^ after 50 cycles).^71^, ^72^ Recently, Bai et al. prepared ultrathin MoS_2_ nanosheets vertically grown on the surface of carbon nanospheres, showing the good mechanical properties of the materials. The reversible capacity of the MoS_2_ nanocomposites maintained a capacity of 650 mAh g^−1^ after 300 cycles, which is attributed to the exceptional robust structural stability to buffer the large volume changes during cycles and the reduction of the diffusion energy barrier of Li^+^ in the lithiation/delithiation processes.^73^

Soft polymer as a template for MoS_2_ nanosheets is another novel way to prepare a 0D sphere, and plays an important role in providing a special dual effect to prevent the aggregation of MoS_2_ nanosheets and absorb the discharge byproduct Li_2_S. For instance, Zheng and Qu et al. loaded MoS_2_ nanolayers on the monodisperse sulfonated polystyrene (SPS) microspheres (served as both the sacrificed soft template and the carbon source) and in situ converted SPS to amorphous carbon after annealing at 800 °C, finally achieving the monodisperse C@MoS_2_ microspheres.^70^ The C@MoS_2_ microspheres with a diameter of 540 nm exhibited uniform ultrathin nanosheets and an interlayer spacing of 0.63 nm, slightly larger than the theoretical value of 0.62 nm as shown in Figure 3e,f. Compared to the bulk MoS_2_, C@MoS_2_ microspheres maintained a capacity of 652 mAh g^−1^ after 100 cycles in Figure 3g, which demonstrated the structural superiority of C@MoS_2_ microspheres. The good cycling performance of C@MoS_2_ microspheres is associated with the interstices between ultrathin MoS_2_ nanosheets and the quasi‐hollow interior on buffering volume changes, and the porous carbon cores on absorbing the discharge byproduct of lithium sulfide. The well‐preserved morphologies of C@MoS_2_ microspheres after 100 cycles can fully validate the hypothesis, which also provides a new idea for MoS_2_‐based materials to achieve good electrochemical performance of LIB. Additionally, for the first tmie, Lou and co‐workers synthesized highly uniform Mo‐glycerate solid spheres via a solvothermal process and the precursor can be easily in situ converted to hierarachical MoS_2_ hollow spheres (HNS) by the subsequent sulfidation reaction, as shown in Figure 3h–i.^74^ The as‐prepared MoS_2_ HNS exhibited a high capacity of 1100 mAh g^−1^ at 0.5 A g^−1^, and long discharge platform in Figure 3j. The product also showed good rate capacities with high capacities at different current densities and stable cycling performances (100 cycles) in Figure 3k,l, which was much better than MoS_2_ solid nanospheres. That is ascribed to the unique hierarchical hollow structure, high surface area, and uniformly distributed structural strains.

Moreover, the electrochemical performance of MoS_2_ sphere also varied with the calcination temperature and the layer distance. According to K. S. Kumar's study, it was found that the MoS_2_ nanoparticle, prepared using supercritical ethanol with a short reaction time, was more likely to perform as a nanoplate structure and it was quite sensitive to the calcination temperature.^75^ This kind of structure was porous and used as an anode for LIBs. The following study confirmed when the calcination temperature was increased from 500 °C to 800 °C, as shown in the Figure 3m,n, the morphology had a great change with the increase of annealing temperature and resulted in the decrease of layer distance from 0.68 nm to 0.61 nm. This downward trend was also detected on Brunner‐Emmet‐Teller (BET) surface area. With the low temperature (500 °C) of the carbonaceous, the initial capacity of MoS_2_ nanoparticles reached to 1342 mAh g^−1^ but the electrodes prepared at high temperature (800 °C) exhibited better cycling performance, as shown in Figure 3o.^59^, ^75^, ^76^

**Figure 4:**
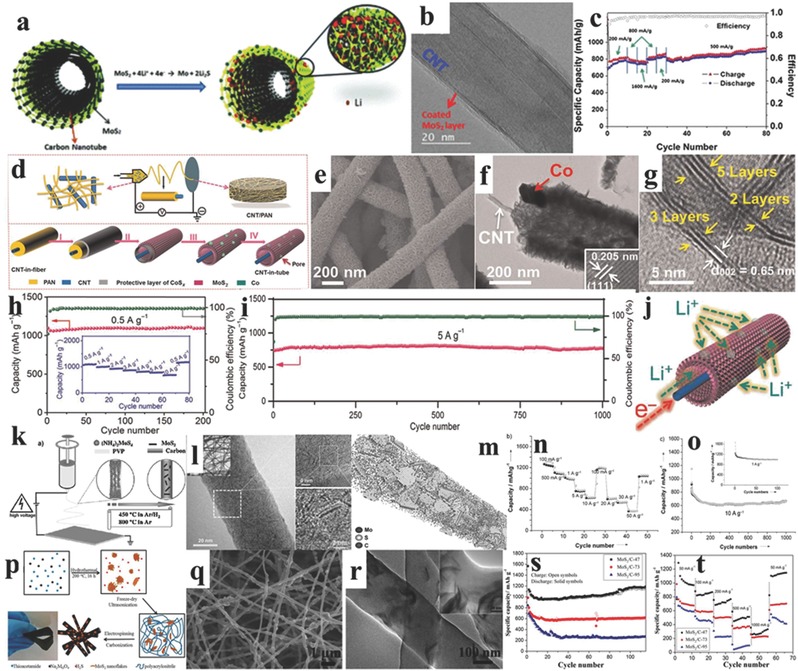
a) The scheme of the MoS_2_/carbon nanotube (MoS_2_/CNT) and reactions with lithium ions. b) HRTEM images of MoS_2_/CNT. c) The rate capabilities of MoS_2_/CNT. Reproduced with permission.^77^ Copyright 2015, Royal Society of Chemistry. d) Schematic illustration of the synthesis of the CNT‐wired hierarchical MoS_2_ tubular structures (CNT/MoS_2_ nanohybrid). Reproduced with permission^80^ Copyright 2016 AAAS. e–g) SEM image, TEM and HRTEM image of CNT/MoS_2_ nanohybrid with layered distance of 0.65 nm. h,i) The rate capacity and cycling performance of CNT/MoS_2_ nanohybrid. j) Schematic of transport paths for Li^+^ ions and electrons in the CNT/MoS_2_ tubular structure. k) The Schematic illustration of the electrospinning process to prepare single‐layered MoS_2_‐carbon nanofiber composites. l) The TEM and HRTEM images of MoS_2_‐carbon nanofiber, showing the short and one‐layer MoS_2_ materials. m) Schematic representation based on TEM modeling studies to demonstrate the unique morphology of such a composite. n,o) The rate capacity and cycling performance of MoS_2_‐carbon nanofiber.^83^ p) The scheme of preparation of MoS_2_/carbon nanofiber composite by the electrospinning process. q,r) SEM and TEM images of MoS_2_/carbon nanofiber composite. s,t) The cycling performances of MoS_2_/carbon nanofiber composite with different weight ratios. Reproduced with permission.^84^ Copyright 2014, American Chemical Society.

Compared to the nanospheres, the 1D materials (nanotubes/nanofibers) have more potential to be applied as electrodes for LIBs because of their high flexibility, one dimensional electron‐transferring channel, and the multiple/interconnected mechanical properties. There are three main matrixes to prepared 1D MoS_2_ nanotube‐like materials, including carbon nanotubes, carbon nanofibers, and metal oxide nanotubes. The MoS_2_ nanosheets can be uniformly wrapped on the surface of CNTs and exhibit enhanced electrochemical performance. For example, Lee and co‐workers used a unique microwave irradiation technique to prepare the cylindrical nanostructure of the MoS_2_‐CNT composite using sulfur and molybdenum chloride as raw materials, and solved the problem of non‐intimate contact between active MoS_2_ and carbonaceous materials, which can be seen in **Figure** **4**a. The cylindrical nanostructure of MoS_2_‐CNT composite is confirmed using high‐resolution transmission electron microscopy (HRTEM), as shown in Figure 4b, with an interlayer distance of 0.61 nm. The corresponding rate capability is shown in Figure 4c, where the capacity increases from 822 mAh g^−1^ to 984 mAh g^−1^ at 200 mA g^−1^ after 10 cycles and the capacity is maintained at 670 mAh g^−1^ at 1600 mA g^−1^. The good electrochemical performances of MoS_2_‐CNT composite are ascribed to the synergistic effects between the cylindrical nanostructured MoS_2_ materials and CNT, and the prevention of aggregation by the unique structure and the high‐conductivity of CNT to improve the conductivity of insulating MoS_2_ materials for LIBs.^77^ A similar strategy was applied by Srivastava and co‐workers and different weight ratios of MoS_2_ and CNT (3:1 to 1:2) were discussed andenhanced electrochemical performances are demonstrated.^78^ Ren et al. used a facile self‐assembly hydrothermal and annealing treatment method to synthesize a MoS_2_/CNT hybrid for LIBs. The few layer MoS_2_ nanosheets were homogenously and vertically grown on the surface of CNTs, thus forming hierarchical nanostructures. The specific capacity of the hybrid reached 1293 mAh g^−1^ when the current density was 200 mA g^−1^.^79^

Another innovation using in situ grown CNTs in hollow carbon nanofibers supporting MoS_2_ ultrathin nanosheets is recently reported by Lou et al.^80^ A 1D CNT/MoS_2_ nanohybrid with the tubular structure was prepared using an electrospinning method with the carboxylic group functionalized CNT as the precursor and polyacrylonitrile (PAN) as the sacrificial template after coating an ultrathin MoS_2_ nanosheets on the outlayer; this can be seen in Figure 4e. The TEM and HRTEM images in Figure 4f,g show the ultrathin MoS_2_ nanosheets on the surface (interlayer distance of 0.65 nm) and the typical tubular structure with CNT as the core and PAN as the shell. The CNT/MoS_2_ nanohybrid electrode delivered a high capacity of ≈1100 mAh g^−1^ at 0.5 A g^−1^ with no obvious capacity decay in 200 cycles, and exhibited an exceptional rate capability up to 10 A g^−1^. Moreover, the CNT/MoS_2_ nanohybrid electrode retained a remarkably reversible capacity of ≈800 mAh g^−1^ at a current density of 5 A g^−1^ after 1000 cycles as shown in Figure 4i. The exceptional electrochemical capacities of the CNT/MoS_2_ nanohybrid were associated with the well‐preserved 1D tubular structure and the fast charge transfer of lithium ions via CNT and tubular MoS_2_, as shown in Figure 4j, indicating that the unique 1D tubular shape and core–shell structure with high conductivity is critical for enhancing the electrochemical performance.

The electrospinning technique has the advantages of low price, being easy‐to‐scale‐up approach, easy operability, and being a versatile technique to gain nanofibers for various functions. Through electrospinning, carbon nanofibers can be coated by MoS_2_ nanocrystals and keep the nanofibers as thin as 100–150 nm in diameter, which can fully encapsulate different active materials for LIBs.^81^, ^82^ Using the electrospinning technique Zhu et al. prepared 1D in situ formed single‐layered MoS_2_‐carbon nanofiber composites as shown in Figure 4k.^83^ The low‐ and high‐magnification TEM images in Figure 4l show that the monolayer MoS_2_ with a length of 4 nm was homo­genously dispersed in the tubular carbon nanofiber. The high conductivity of the carbon nanofiber provided both an electron transfer channel and a well‐connected matrix for monolayer MoS_2_ nanoplates, which is shown in the 3D TEM model in Figure 4m. The 1D single‐layer MoS_2_‐carbon nanofiber composites exhibited exceptional rate capacities (Figure 4n), delivering the specific capacities of 1095, 986, and 974 mAh g^−1^ at current densities of 0.5, 1, and 50 A g^−1^, respectively. The materials also showed good cycling performances, maintaining a high capacity of 1007 mAh g^−1^ at 1 A g^−1^ and 661 mAh g^−1^ at 10 A g^−1^, respectively. Moreover, the 1D single‐layered MoS_2_‐carbon nanofiber composites were also used as an anode for sodium ion batteries and exhibited enhanced rate capacities and cycling performances. Similarly, Lu and co‐workers used the pre‐prepared thin MoS_2_ nanoflakes and synthesized the 1D tubular MoS_2_/carbon nanofiber via the electrospinning technique with PAN as the carbon source in Figure 4p, and the morphology of MoS_2_/carbon nanofiber with the MoS_2_ content of 47 wt% is presented in Figure 4q–r, illustrating the uniformly distributed MoS_2_ nanoflakes in carbon nanofibers. The electrochemical performance of different MoS_2_ content (47%, 73%, and 95%) in carbon nanofibers is presented in Figure 4s,t, demonstrating that the MoS_2_/C‐47 maintains the most stable cycling performance and rate capacity.^84^ Moreover, Wang et al. have reported an intensive investigation of different nanofibers for energy storage applications.^85^, ^86^ According to the report of Hu et al., a kind of carbon nanofiber composites made up of MoS_2_ nanocrystal and carbon nanofiber showed excellent cycling performance as an anode for LIBs.^87^ In 2015, Kong and co‐workers started a comprehensive investigation into the electrochemical performance of MoS_2_, which is fostered in three different kinds of nanofibers: solid carbon nanofibers (SNFs), hollow carbon nanofibers (HNFs), and porous carbon nanofibers (PNFs).^88^ They found a phenomenon that the MoS_2_ was grain stacking together on the surface of SNFs and HNFs very severely while this phenomenon seldom existed on the inside surface of PNFs.^88^ Furthermore, the electrochemical performance of MoS_2_ sheets, which are grown on the porous carbon nanofibers, was examined at different current densities. The specific capacity of MoS_2_/PNFs reached 1210 mAh g^−1^ at 100 mA g^−1^, which was higher than the previously reported value (1290 mAh g^−1^ at 50 mA g^−1^).^89^ Therefore, the MoS_2_/PNF nanocomposites with MoS_2_ nanosheets fabricated on nanofibers are an excellent representation as the anode for LIBs. Miao et al. fabricated porous carbon nanofiber‐MoS_2_ composites with a core–shell structure for the anode of LIB.^90^ The X‐ray diffraction (XRD) pattern shows three distinct diffraction peaks (2θ = 13.9°, 33.4°, and 58.9°), which relpresent the corresponding (002), (100), and (110) planes, respectively. The typical shift of the (002) diffraction peak reflects the unique standard hexagonal 2H‐MoS_2_ structure, which facilitates the diffusion of lithium ions. The capacity can reach 1532 mAh g^−1^ at 0.1 C. About 76.5% capacity remained because of the formation of the solid electrolyte surface (SEI) layers and the trapping of lithium in the lattice and defects of MoS_2_ after the first cycle. Then, the capacity retention ratio reached 100% in the subsequent cycles.^90^ Qu et al. reported a mature method of electrospinning technology to modify a kind of CNTs@MoS_2_ hybrid nanocomposite.^91^ The as‐prepared nanocomposites showed a specific capacity of 1027 mAh g^−1^ and only a slight capacity decay was found after 100 cycles. However, the conducting polymer composites are not stable during cycling, which is ascribed to the non‐uniform dispersion and the relatively low conductivity of MoS_2_.

**Figure 5:**
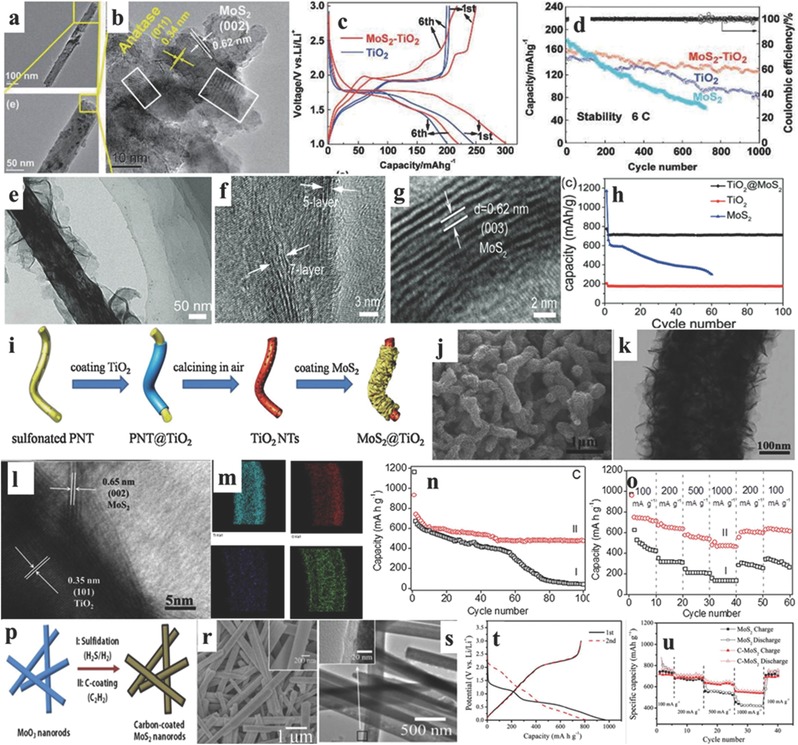
a,b) Low and high magnification images of MoS_2_‐TiO_2_ nanofibers. c‐d) The charge/discharge profiles of TiO_2_ and MoS_2_‐TiO_2_ nanofibers, and the comparison of cycling performances of the MoS_2_, TiO_2_ and MoS_2_‐TiO_2_ nanofibers. Reproduced with permission.^93^ Copyright 2015, WILEY‐VCH Verlag. e–g) TEM and HRTEM images of TiO_2_@MoS_2_ nanobelt with layer distance of 0.62 nm. h) Cycling performance of MoS_2_, TiO_2_ and TiO_2_@MoS_2_ nanobelts at the current density of 100 mA g^−1^. Reproduced with permission.^94^ Copyright 2014, Royal Society of Chemistry. i) The schematic illustration of the preparation procedure of MoS_2_ nanosheets@TiO_2_ nanotube hybrid materials. j–l) The SEM and TEM images of MoS_2_@TiO_2_, showing the lattices of MoS_2_ and TiO_2_, respectively. m) Elemental mapping images of MoS_2_@TiO_2_, showing individual elements in MoS_2_@TiO_2_ hybrids. n,o) The cycling performance and rate capability of MoS_2_@TiO_2_ hybrids. Reproduced with permission.^95^ Copyright 2014, Royal Society of Chemistry.p) The scheme of C‐MoS_2_ nanorods. r‐s) The SEM and TEM images of C‐MoS_2_ nanorods. t,u) The charge/discharge profiles and rate capability of C‐MoS_2_ nanorods. Reproduced with permission.^97^ Copyright 2012, Elsevier B.V.

Moreover, the inorganic material MoS_2_ combined with organic materials is a new protocol to develop electrodes for LIBs. A good illustration of MoS_2_‐PANI nanowires (MoS_2_ 66.7%: PANI 33.1%) composite is shown as a well‐designed hierarchical nanostructure under mild conditions. That delivered a high reversible specific capacity of 1063.9 mAh g^−1^ at 100 mA g^−1^ with a capacity retention ratio of 90.2% after 50 cycles.^92^ Metal oxides nanowires, such as TiO_2_ nanotube/nanofiber, MoO_3_ nanorods, or MnCO_3_, can be applied as a substrate or raw material to prepared 1D MoS_2_ nanohybrids with enhanced electrochemical performance. For example, compared to carbon nanofibers, mesporous TiO_2_ nanofibers exhibit outstanding flexibility and plasticity features, which can be used as main bone to support MoS_2_. For example, Ying et al. used mesoporous TiO_2_ nanofibers as templates to prepare MoS_2_‐TiO_2_ nanofibers by in situ vulcanizing the (NH_4_)_6_Mo_7_O_24_·4H_2_O precursor by H_2_S. The TEM and HRTEM images are shown in **Figure** **5**a,b, showing the anatase TiO_2_ (011) plane inside layer and MoS_2_ (002) as the outside layer. The typical charge/discharge profiles of TiO_2_ and MoS_2_‐TiO_2_ nanofibers are shown in Figure 5c, demonstrating that the MoS_2_‐TiO_2_ nanofibers delivered a higher capacity (301 mAh g^−1^) than bare TiO_2_ (247 mAh g^−1^) and a longer discharge platform. The MoS_2_‐TiO_2_ nanofibers also exhibited stable cycling performance for 1000 cycles at 6C, which was much better than bare TiO_2_ and MoS_2_ electrodes, as shown in Figure 5d. The exceptional electrochemical performance is probably due to the synegitic effect between the mesoporous TiO_2_ structure and the homogenously distributed MoS_2_ nanosheets.^93^ Similarly, Wang and co‐workers synthesized few‐layered MoS_2_‐coated TiO_2_ nanobelts (TiO_2_@MoS_2_) via the hydrothermal method andthe 1D structure is shown in Figure 5e. The corresponding HRTEM images in Figure 5f,g show the few layers of MoS_2_ on the TiO_2_ nanobelt and the layer distance of 0.62 nm. The TiO_2_@MoS_2_ hybrid maitains a high capacity of 710 mAh g^−1^ after 100 cycles at 100 mA g^−1^, which is four times higher than that of bare TiO_2_ nanobelt and three times higher than that of MoS_2_. That is related to the integerity of 1D nanostructure after long‐term cycles and the ultrathin MoS_2_ nanosheet shortening the diffusion paths of Li^+^ ions.^94^ Moreover, Xin et al. synthesized MoS_2_@TiO_2_ nanocomposites with thin MoS_2_ nanosheets grown on TiO_2_ nanotubes using a sol×gel method, as shown in Figure 5i.^95^ The MoS_2_ nanosheets are homogenously attached to the surface of TiO_2_ nanotubes, which can be observed in the SEM and HRTEM images in Figure 5j–l. The corresponding elemental mapping images in Figure 5m vividly show the well‐distributed MoS_2_ on TiO_2_ nanotubes. The initial reversible capacity of this material was 931 mAh g^−1^ and it remained 578 mAh g^−1^ after 100 cycles, as shown in Figure 5n. The exceptional rate capability in Figure 5o illustrates that the MoS_2_@TiO_2_ is enhanced in electrochemical performance compared to the bare MoS_2_ nanosheets. Another example of a 1D MoS_2_ material is the typical hierachical hollow MoS_2_ nanotubes that are produced by a solvothermal reaction of Na_2_MoO_4_·2H_2_O, MnCl_2_·4H_2_O, and (NH_4_)_2_CS, followed by etching the MnCO_3_ nanotubes.^96^ The capacity stayed as high as 727 mAh g^−1^ after 100 cycles at a current density of 100 mA g^−1^. The impressive electrochemical performance is possibly attributed to the special hierarchical surface and hollow tube structure by post‐mortem analysis. Zhang et al. used MoO_3_ nanorods as the precursor and in situ sulfidation by H_2_S/H_2_, and then added an additional carbon layer via a chemical vapor deposition (CVD) method to synthesize carbon‐coated MoS_2_ (C‐MoS_2_) nanorods as shown in Figure 5p. The corresponding SEM and TEM images in Figure 5r,s show the homogenous 1D C‐MoS_2_ nanorods. The as‐prepared carbon‐coated MoS_2_ nanorods exhibited the typical charge/discharge profiles of C‐MoS_2_ nanorods (Figure 5t) and maintained a high capacity of 621 mAh g^−1^ at the current density of 200 mA g^−1^ after 80 cycles (Figure 5u). According to the experimental results, the carbon layer plays an important role in stabilizing the cycling performance and enhancing the conductivity of the electrode.^97^

#### 2.1.2 Two Dimensional MoS_2_

**Figure 6:**
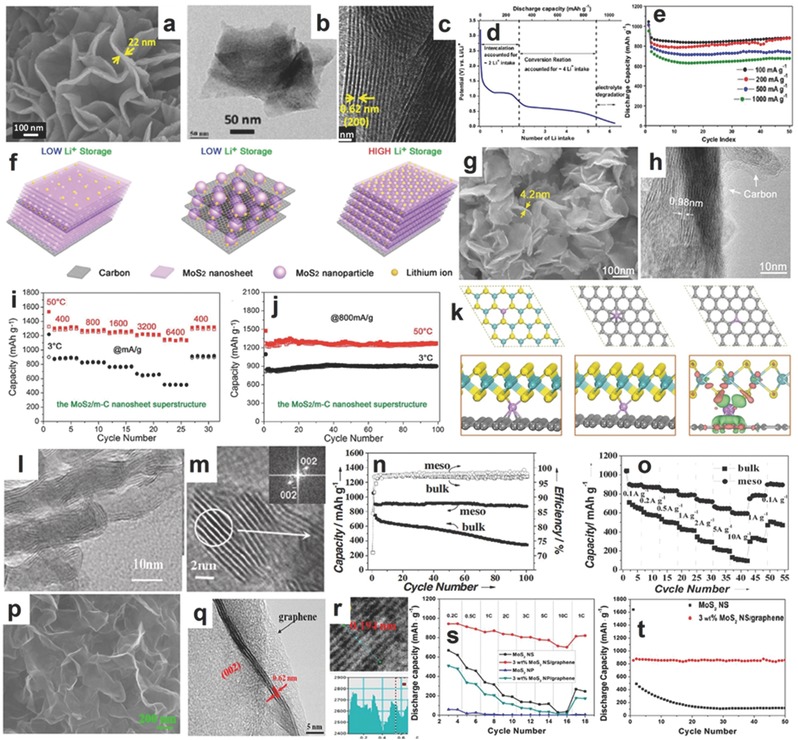
a–c) The SEM, TEM and HRTEM images of MoS_2_ nanowall, respectively. d,e) The discharge profile, cycling performance and rate capability at different current densities of MoS_2_ nanowall. Reproduced with permission.^101^ Copyright 2013, American Chemistry Society. f) The scheme of lithium storage at MoS_2_/carbon atomic interface. g‐h) The SEM, TEM and TEM elemental mapping images of MoS_2_/carbon nanosheets, respectively. i,j) The rate capability and cycling performance of MoS_2_/carbon nanosheets at 3 °C and 50 °C. k) The theoretical calculation for the most stable configuration of lithium ion absorption in different systems. Reproduced with permission.^102^ Copyright 2015 WILEY‐VCH. L,m) The TEM and HRTEM images of mesoporous MoS_2_ nanosheets with a layer distance of 0.66 nm. n,o) The cycling performance and rate capacities for mesoporous MoS_2_ and bulk MoS_2_, respectively. Reproduced with permission.^103^ p–r) The SEM, HRTEM, and fast Fourier transform (FFT) pattern of MoS_2_/graphene composites. s,t) The rate capability and cycling performance of MoS_2_/graphene composites and bare MoS_2_. Reproduced with permission.^104^ Copyright 2013, American Chemical Society.

In recent years, 2D materials have attracted tremendous attention for their unique properties. Few layered MoS_2_ has a good band gap (≈1.8 eV) and its unique structure gives the convenience of the intercalation and transportation of Li^+^ ions. To fully magnify this edge, 2D MoS_2_ is often synthesized with amorphous carbon, conductive polymers, and metal oxide decoration to do the surface‐treatment.^88^, ^91^, ^98^, ^99^, ^100^ Mitra and co‐workers, prepared the 2D MoS_2_ nanowalls via a two‐step reaction with freshly prepared H_2_S and hydrothermal post‐treatment. It was also reported that the use of carboxymethyl cellulose (CMC) with a strong interaction for the conversion reaction electrode exhibits a good mechanical binding effect and stability compared to PVDF. The SEM and TEM images in **Figure** **6**a–c show the typical layered structure and the a layer distance of 0.62 nm.^101^ The typical discharge profile in Figure 6d exhibits three main lithiation processes, including the intercalation of Li^+^ ions in the interlayer space at 1.1 V with around 1.8 Li^+^ intake, the conversion reaction with Li^+^ ions at 0.6 V with around 4 Li^+^ ions consumption, and electrolyte decomposition reaction or other reactions. The 2D MoS_2_ nanowalled electrode with CMC as the binder delivered a high capacity of 1047 mAh g^−1^ and maintained around 880 mAh g^−1^ after 50 cycles at 100 mA g^−1^ (Figure 6e). The MoS_2_ nanowalled electrode also exhibited superior rate capacity at different current densities, which is attributed to the 2D nanowalled structure and high surface area as well as the connection of the CMC binder.

As the special lithiation process of MoS_2_ is highly dependent on the structural arrangement and interface contact with a conductive mediator, such as mesoporous carbon nanosheets, mesoporous carbon, or graphene, the ideal structure for MoS_2_ materials was proposed by Li et al. and is shown in Figure 6f,^102^ where the single‐layer MoS_2_ nanosheet is atomic interfacial contacted with a single‐layer carbon nanosheet. The layered MoS_2_/mesoprous carbon (MoS_2_/m‐C) is prepared by the oleic acid (OA) pre‐functionalized MoS_2_ nanosheet reacting with dopamine and annealed at 850 °C for 2 h. The 2D nanocomposites piled by single layer MoS_2_ and mesoporous carbon was produced as an anode for LIBs. The ideal atomic interface contacts between two kinds of layers promising the maximization of synergistic interaction. The blue shift of peaks at 8.9° and 17.9° in the XRD pattern was attributed to the insertion of the OA molecules. Figure 6g,h show the typical layered structure with carbon as the interlayer mediator, and the TEM elemental images demonstrate the well‐overlapped element distribution. The layered MoS_2_/m‐C nanocomposites delivered a high capacity of 1113 mAh g^−1^ at 400 mA g^−1^ and maintained 1023 mAh g^−1^ after 500 cycles. Additionally, the MoS_2_/m‐C hybrid also showed improved rate capability compared to bare MoS_2_ and graphene. As far as the electrochemical performance of MoS_2_/m‐C hybrid at extreme temperature, the hybrid exhibited much better rate capability and cycling performances at high temperature (50 °C, mimicking a desert environment) than in a cool environment (3 °C), as shown in Figure 6i,j. Furthermore, the theoretical calculations using first‐principle density functional theory (DFT) illustrated the enhancement of Li ion intercalation in the MoS_2_/G (or MoS_2_/G/MoS_2_) at the interface because of the synergistic interaction as shown in Figure 6k, demonstrating that the MoS_2_ layer is bonded with carbon nanosheets via van der Waals interactions. Wang et al. synthesized highly ordered mesoporous MoS_2_ using a vacuum‐assisted impregnation method_,_ which has an expanded spacing of the (002) crystal plan that can be used in the LIBs (Figure 6l,m). The cycling performance of highly ordered mesoporous MoS_2_ and bulk MoS_2_ as a comparison is presented in Figure 6n, illustrating that highly ordered mesoporous MoS_2_ shows high specific capacity (1052 mAh g^−1^) and good cycling performance over 100 cycles. Moreover, the highly ordered mesoporous MoS_2_ anode shows good rate capability in Figure 6o (a high specific capacity of 608 mAh g^−1^ at the current density of 10 A g^−1^), which is much higher than the bulk MoS_2_ anode.^103^ The exceptional electrochemical performance of highly ordered mesoporous MoS_2_ is related to the homogenously distributed nanopores, high surface area, and the high pore volume, which are beneficial to the lithium transport and electrolyte diffusion. The intrinsic property of highly ordered mesoporous MoS_2_ also provides the short crystalline pathways for lithium ions, low activation energy phase, and wide layer distance at (002) plane. The combination of MoS_2_ sheets and graphene is another approach to avoid materials aggregation and enhance the conductivity of MoS_2_. Park et al. prepared ultrathin MoS_2_/graphene composites using the hydrothermal reaction and simultaneously reduced the graphene oxide with NaOH. The SEM and TEM images in Figure 6p,q illustrate the well‐distributed MoS_2_ in graphene (3%) nanosheets,^104^ and the HRTEM image in Figure 6r shows the few layers of MoS_2_ with an interlayer of 0.62 nm at (002) plane and the typical (100) plane with a lattice of 0.194 nm. The comparison of rate capacity and cycling performance in Figure 6s,t demonstrates that the MoS_2_/graphene (3%) composite is much better than that of bare MoS_2_ or graphene, respectively, indicating that the introduction of graphene sheets can effectively absorb the inner mechanical strain, confine the volume changes, and enhance the conductivity of MoS_2_ nanosheets.

Inspired by an adhesive protein that is usually found in mussels, biologic materials have been used to prepare various materials with special structures. Polydopamine (PD) has recently shown great possibilities for the surface modification of organic and inorganic materials via polymerization. As a good self‐assembly agent and a carbon precursor, PD can be absorbed on exfoliated MoS_2_ monolayers through electrostatic interactions and then in situ turned to graphitic carbon through a pyrolysis process.^105^ After being coated with PD and graphited in inert gas, ultrathin MoS_2_@N‐doped nanocomposites with a sandwiched structure can be obtained. Hu synthesized loose carbon‐coated MoS_2_ sheets via a one‐pot hydrothermal synthesis method, which exhibited a high capacity of 1419 mA h g^−1^ at 0.1 A g^−1^ and the capacity remained 80% after 50 cycles.^106^ Cho et al. prepared MoS_2_ nanoplates with a graphene‐like layered structure and a thickness of 30 nm via a simple, scalable, and one‐pot reaction method, showing a high reversible capacity of 700 mAh g^−1^ at 50 C. Additionally, a demonstration of a full battery (LiCoO_2_/MoS_2_) exhibited a stable cycling performance.^179^

**Figure 7:**
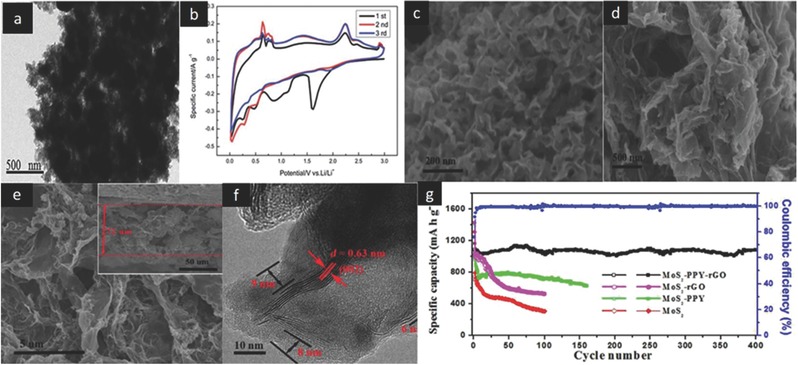
TEM images of the Sn/MoS_2_/C composite (a) and the CV curve of this material as anode (b). Reproduced with permission.^107^ Copyright 2016, Royal Society of Chemistry. The SEM image of the MoS_2_ nanosheet (c). SEM image of MoS_2_‐PPY‐rGO (d,e). TEM images of MoS_2_‐PPY‐rGO (f) and cycling performances of MoS_2_‐PPY, MoS_2_‐PPY‐rGO, MoS_2_‐rGO, and MoS_2_ materials at the current density of 200 mA g^−1^ (g). Reproduced with permission.^99^ Copyright 2015, Elsevier B.V.

Apart from the carbon layer overlapped structure with MoS_2_, some metal nanoparticle permeating MoS_2_ materials are also demonstrated as an effective approach to enhance the electrochemical performance of LIBs. For example, combined with MoS_2_, the drawbacks of bare tin with a fast capacity fade and poor rate capability can be easily avoided, while its advantages such as low toxicity and high capacity still remain.^107^ **Figure** **7**a,b show the TEM image and the cyclic voltammetry (CV) curve of the Sn/MoS_2_/C nanomaterial. The Sn/MoS_2_/C anode delivered a high capacity of 1050 mA h g^−1^ at 100 mA g^−1^, which is attributed to the common contributions of Sn and MoS_2_ for the achieved capacity. The co‐existence of Sn nanoparticles can promote the growth of stable solid electrolyte interphase films and prevent the rupture of SEI during the charging process. At the same time, MoS_2_ nanosheets can suppress the aggregation of the Sn nanoparticles and keep them in a homogenous distribution state.^107^ Xie et al. synthesized flower‐like polypyrrole (PPy) coated on the reduced graphene oxide (rGO) supported MoS_2_ nanosheets (MoS_2_‐PPy‐rGO) via the chemical bonding.^99^ The typical morphologies of MoS_2_‐PPy‐rGO nanocomposites are shown in Figure 7c–f. The initial discharge capacity delivered a high capacity of 1428 mAh g^−1^ at a current density of 200 mA g^−1^ and there was about 1070 mAh g^−1^ remaining after 400 cycles (Figure 7g), which was attributed to the synergetic effect of flexible rGO and flower‐like PPy‐MoS_2_ nanosheets. Wang et al. made a brief investigation about the impact of various guest interactions on the MoS_2_ nanosheets.^108^ Guest species include amorphous carbon, dopamine, polyvinyl pyrrolidone, (PVP), and ethylene diaminetrimolybdate (EDA). All of the composites are bridged into MoS_2_ slabs by chemical bonding and the interactions of structure stabilities.

#### 2.1.3 Three Dimensional MoS_2_

**Figure 8:**
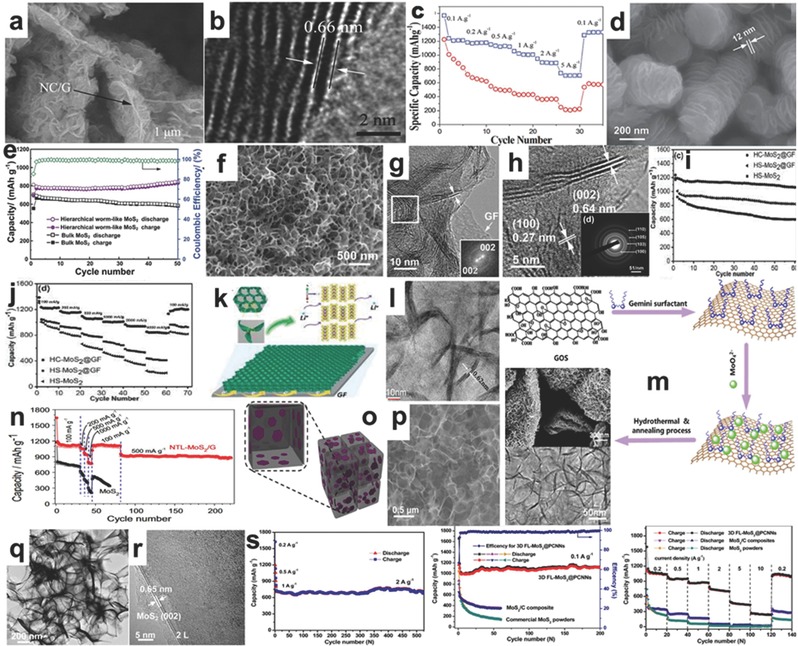
a,b) The SEM and HRTEM images of MoS_2_/NC/G hybrid. c) The rate capacities of MoS_2_/NC/G hybrid and bare MoS_2_. Reproduced with permission.^109^ Copyright 2014, Royal Society of Chemistry. d) The SEM image of worm‐like MoS_2_ materials. e) The cycling performance of worm‐like MoS_2_ materials and bulk MoS_2_.^110^ f‐g) The SEM, TEM and HRTEM images of HC‐MoS_2_@GF, respectively. i,j) The cycling performance and rate capacity of HC‐MoS_2_@GF and HS‐MoS_2_. k) The scheme of the additional active edge sites and the fast electron transportation in HC‐MoS_2_@GF.^111^ i–m) The TEM image of MoS_2_/graphene, and the diagram of the synthesis process, the FESEM and TEM images of MoS_2_/graphene nanocomposites. n) The rate capability of MoS_2_/graphene composites. Reproduced with permission.^118^ Copyright 2014, Elsevier B.V. o–r) The scheme, SEM, TEM and HRTEM images of 3D FL‐MoS_2_@PCNNs composites, respectively. s) The cycling performances and the rate capabilities of 3D FL‐MoS_2_@PCNNs composites, commercial MoS_2_ and MoS_2_/C composite, respectively. Reproduced with permission.^119^ Copyright 2015, American Chemistry Society.

The 3D MoS_2_ nanocomposites consisting of flower‐like, worm‐like, honeycomb‐like, and other special morphologies exhibit exceptional features in terms of both mechanics and electrochemical performance. Flower‐like and nitrogen–doped, the polypyrrole (PPy)‐MoS_2_/NC/G hybrids shown inthe SEM and HRTEM in **Figure** **8**a,b, exhibited enhanced electrochemical performance compared to bare MoS_2_. The MoS_2_/NC/G hybrids delivered a high rechargeable capacity of 1570.6 mAh g^−1^ at the condition of 0.1 A g^−1^ and superior rate capability, which were higher than that of 2D bare MoS_2_ materials due to the well‐connected and open framework structure of the flower‐like and nitrogen‐doped PPy‐MoS_2_/NC/G hybrids, as shown in Figure 8c.^109^ Materials with a worm‐like structure have flexible and self‐connected features that can resist large mechanical stress and show good integrity during long‐term cycles. Wang et al. successfully synthesized worm‐like MoS_2_ nanosheets using potassium sodium tartrate as an agent using a facile treatment, and the typical worm‐like morphology is shown in Figure 8d. The capacity of the worm‐like MoS_2_ nanosheets was 845 mAh g^−1^ after 50 cycles at 100 mA g^−1^, as shown in Figure 8e, and maintained a specific capacity of 698 mAh g^−1^ at higher current rate of 500 mA g^−1^ after 100 cycles, which is associated with the large surface area (providing more exposed active sites and a large contact area), the free space between neighboring nanosheets (buffering the large mechanical stress stemming from the large volume changes due to the conversion reaction), and the self‐interconnected structure (avoiding electrode aggregation).^110^

Moreover, the honeycomb‐like structure with the interconnected net and robust mechanical property has recently attracted more attention. For example, the honeycomb‐like MoS_2_ nanoarchitecture prepared by a facile P123‐assisted solution‐phase approach and loaded into 3D graphene foam (GF) reported by Lin and Shen et al.,^111^ exhibited a high specific surface area and electrochemical stability, showing a high capacity of 1235.3 mAh g^−1^ at 200 mA g^−1^ and retaining 85.8% of the reversible capacity after 60 cycles. The corresponding SEM, TEM, and HRTEM are shown in Figure 7f–h with a layer distance of 0.64 nm and 0.27 nm at different phase orientations. The cycling performance and rate capability of the 3D honeycomb‐like MoS_2_@GF materials are presented in Figure 8i,j. The scheme in Figure 8k shows that the additional active edge sites and the fast electron transportation in HC‐MoS_2_@GF contributed to the exceptional rate capabilities and good cycling performances. The synergistic interaction between MoS_2_ and graphene can prevent the severe expansion of MoS_2_ layers during discharge.^112^ Meanwhile, the existence of MoS_2_ can reduce the instinct of wrapping of graphene layers and keep its structured performance.^113^ The homogenous distribution of MoS_2_ in other carbon‐based materials can significantly improve the use efficiency of the active sites. Zhang and co‐workers carried out a specific investigation into the use of graphene and its derivatives and found many new characteristics.^114^ His group also successfully obtained large‐scale, highly crystalline MoS_2_ sheets on insulating substrates.^115^ Compared to other exfoliated MoS_2_ and bulk MoS_2_, the sandwich MoS_2_@N‐doped carbon nanosheets exhibited enhanced lithium storage capability. The specific capacity of the aforementioned nanocomposites reached 1239 mAh g^−1^ and remained at 597 mAh g^−1^ at 10 A g^−1^.^116^ Ye et al. successfully synthesized a similar sandwiched MoS_2_/graphene composite using polydiallydimethylammonium chloride (PDDA).^117^ When the added amount of PDDA was 0.02 mol L^−1^, the corresponding capacity of the as‐prepared materials was 1100 mAh g^−1^ with an enhanced cycling stability, and around 856 mAh g^−1^ remained after 900 cycles.^117^

According to previous publications, the application of various surfactants can help the construction of different nanostructures via the hydrophilic/hydrophobic attractions and the changes of MoS_2_ nanomaterials significantly influence the electrochemical performance. Ma et al. compared the influencevarious types of cationic surfactants on of morphology changes of MoS_2_ nanomaterials, including dodecyltrimethyl ammoniumbromide, octyltrimethyl ammoniumbromide, and tetrabutyl ammoniumbromide.^118^ Moreover, the Gemini surfactants composed of two hydrophobic tails and two hydrophilic heads illustrate the exceptional features of lowering surface tension and organizing the novel morphologies of MoS_2_ nanomaterials. Assisted by a type of N‐dodecylpropylenediamine γ‐diquaternium bromide, the tile‐like MoS_2_/graphene nanocomposites were prepared and are shown in Figure 8l,m. Layers of MoS_2_ (3–6) are homogenously anchored on the surface of the graphene sheets andexhibited high surface area, exposing tremendous active surfaces of MoS_2_ nanosheets. The rechargeable capacity of the nanotile‐like MoS_2_/graphene nanocomposites delivered a high reversible capacity of 1127 mAh g^−1^ at a current density of 100 mA g^−1^ and showed stable cycling performance in 220 cycles and good rate capacities at different current densities, as shown in Figure 8n. The current densities are attributed to the well‐distributed nanotile‐like MoS_2_ nanosheets on the graphene nanosheets.^118^ Another demonstration of 3D porous carbon nanosheet networks used as a matrix to anchor few layered MoS_2_ nanosheets was reported by Zhao and He et al. (Figure 8o).^119^ The NaCl nanocrystal mixed with Mo‐based raw materials is used as a sacrificial template and heated at 750 °C, simultaneously turning MoO*_x_* to MoS_2_ nanosheets on its surface by in situ generated H_2_S in an Ar environment. The processis easily controllable, non‐toxic, low cost, and easy‐to‐scale‐up for industrial production. The corresponding interconnected 3D porous network with ultrathin MoS_2_ nanosheets can be seen in the SEM, TEM, and HRTEM images in Figure 8p–r. When used as anode materials for LIBs, the 3D FL‐MoS_2_@PCNNs materials exhibited a high specific capacity of 1161 mAh g^−1^ at 0.1 mA g^−1^, and a high capacity of 709 mAh g^−1^ at 2 A g^−1^ and retained 95.2% capacity after 520 cycles. Moreover, it also exhibited superior rate capability at different current densities (Figure 8s).^119^ Chen et al. using l‐cysteine as a surfactant prepared the layered MoS_2_/graphene composites for anode materials of LIBs, exhibiting a high reversible capacity of 1100 mAh g^−1^ at 100 mA g^−1^ and good cycling stability.^180^

**Figure 9:**
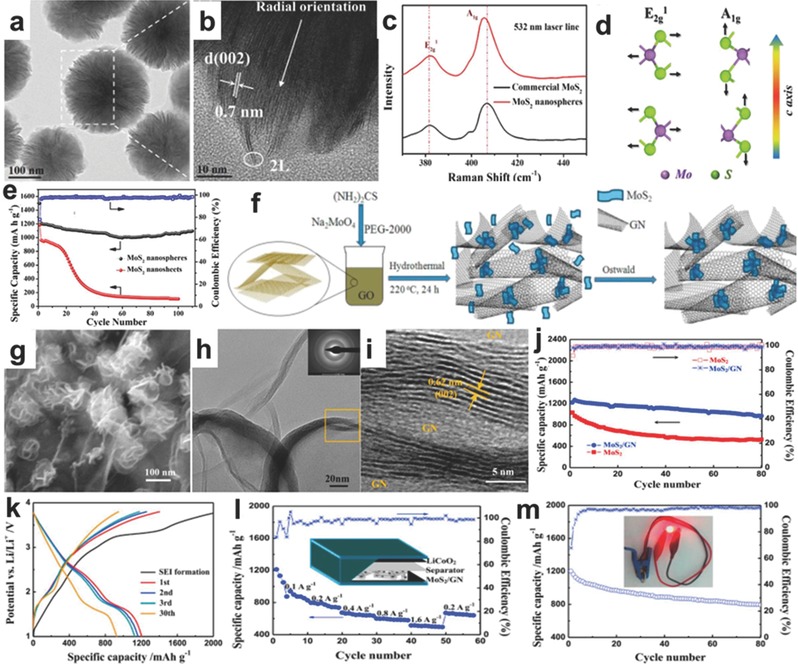
a,b) The SEM and TEM images of 3D radially oriented MoS_2_ nanospheres. c,d) The Raman spectra of MoS_2_ nanospheres and commercial MoS_2_, and the atomic vibration modes of E_2g_^1^ and A_1g_. e) The cycling performance of MoS_2_ nanospheres.^120^ f) Schematic diagrams of the synthesis process g–i) SEM, TEM and HRTEM images of MoS_2_/graphene (j). The cycling performance of MoS_2_/graphene composites. k–m) The charge/discharge profile, rate capacity and cycling performance of the MoS_2_/graphene/LiCoO_3_ full battery with a LED light powered by a full battery. Reproduced with permission.^121^ Copyright 2016, Elsevier B.V.

Zhang et al. synthesized MoS_2_ nanospheres with a 3D radially oriented architecture via a hydrothermal method and the assistance of PVP surfactant, which displayed plate‐like and radially oriented morphology and exceptional mechanical flexibility. The typical radially oriented MoS_2_ nanospheres are shown in **Figure** **9**a,b, demonstrating the larger layer distance of 0.7 nm.^120^ The Raman spectrum is recognized as a reliable diagnostic tool to check the ultrathin nature of MoS_2._ In Figure 9c,d, the spectra confirmed the decreased number of ultrathin MoS_2_ nanospheres to the bulk MoS_2_, and the corresponding atomic vibrational modes illustrated the vibrational directions of the MoS_2_ nanospheres. When employed as the anode of a LIB, the capacity of 3D radially oriented MoS_2_ nanospheres reached 1009.2 mAh g^−1^ at the current density of 500 mA g^−1^ after 500 deep charge and discharge cycles as shown in Figure 9e. Yuan and co‐workers carried out a comprehensive study of the application of 3D hierarchical MoS_2_/graphene materials used as half‐cell and full‐cell anodes in LIBs.^121^ The 3D hierarchical material was prepared by a facile hydrothermal method with the PEG‐2000 as the surfactant. Figure 9f shows the typical synthesis process for the MoS_2_/graphene composites. The morphologies of MoS_2_/graphene composites are presented in Figure 9g–i with the interlayer distance of 0.62 nm in the phase of (002). The MoS_2_/graphene composites delivered a high initial capacity of 1240 mAh g^−1^, and maintained a reversible capacity of 970 mAh g^−1^ as shown in Figure 9j, which is much more stable than the bulk MoS_2_.The MoS_2_/graphene composite was also tested as MoS_2_/GN/LiCoO_2_ full‐cells. The relationship between the capacity and the potential of Li/Li^+^ is shown in Figure 9k. In the full cell, the MoS_2_/graphene composite delivered a high reversible capacity of 1203 mAh g^−1^ and exhibited both the stable rate capability and the favorable cycle stability in Figure 9l,m with an illustration of powering a small light‐emitting diode (LED). This ideal performance is attributed to factors including cross‐linked graphene, well‐crystallized MoS_2_ nanoflakes, and a synergistic effect between two types of materials.^121^

**Figure 10:**
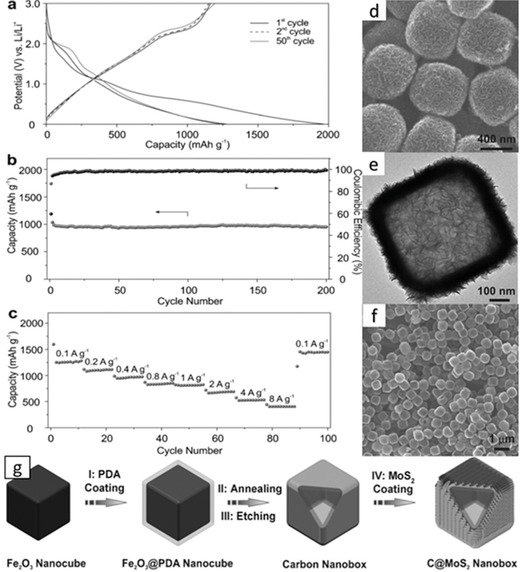
a) Charge and discharge voltage image of the first, second and 50th cycles at the current density 0.1 A g^−1^. b) The relationship between cycling performance and corresponding when the current was 0.4 A g^−1^. c) The rate performance at different current rates. d–f) FESEM images and TEM (e) image of C@MoS_2_ nanoboxes. g)Schematic diagrams of the process of the synthesis of C@MoS_2_ nanoboxes. Reproduced with permission.^122^ Copyright 2015, Wiley‐VCH Verlag.

The morphology of MoS_2_ is easily controlled by surfactants, graphene oxides, functionalized CNTs, and metal–organic frameworks (MOF), which can substantially improve the electrochemical and mechanical properties of MoS_2_ materials. The MOF‐derived carbon framework contains a nitrogen‐doped hybrid structure, with high conductivity and additional lithium ion storage. Yu et al. synthesized a C@MoS_2_ nanobox by fostering the ultrathin MoS_2_ nanosheets on N‐doped carbon shells and tested it on the LIBs. They thought that the nanosheets could provide more active sites for electrochemical reactions. In the first cycle, the charge and discharge capacity of C@MoS_2_ nanoboxes were as high as 1966 mAh g^−1^ and 1164 mAh g^−1^ and only about 59.2% was retained. The large capacity loss was ascribed to the decomposition of the electrolyte and the irreversible lithiation process, the formation of solid electrolyte interface. **Figure** **10** shows the electrochemical performance, field‐emission scanning electron microscopy (FESEM) image,TEM image, and the schematic diagrams of the synthesis of the nanoboxes.^122^

**Figure 11:**
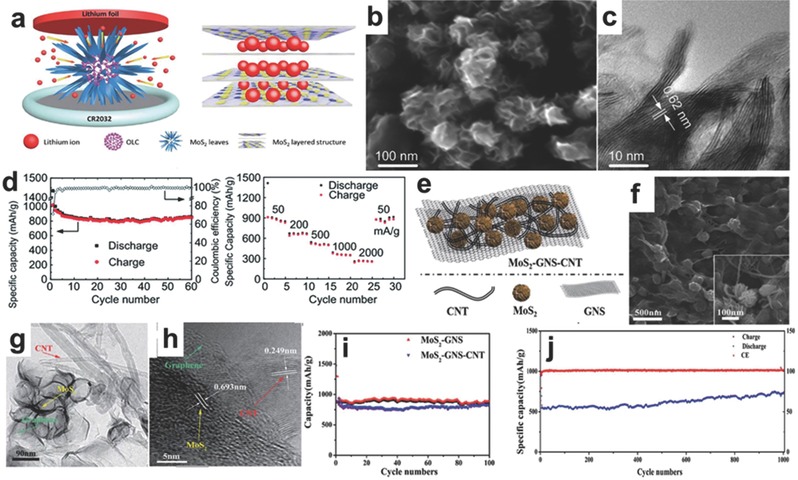
a) Schematic illustration of the pre‐lithiation process, device configuration and the MoS_2_ layered structure with the intercalated lithium ions during the synthesis process. b,c) The SEM and HRTEM image of MoS_2_ nanourchins. d) The cycling performance and rate capacity of MoS_2_ nanourchins. Reproduced with permission.^123^ Copyright 2015, Royal Society of Chemistry. e) The scheme of MoS_2_‐GNS‐CNT hybrid. f‐h) SEM, TEM and HRTEM images of MoS_2_‐GNS‐CNT hybrid, respectively. i,j) The cycling performances of MoS_2_‐GNS‐CNT hybrid and MoS_2_‐GNS composites. Reproduced with permission.^124^ Copyright 2015, Royal Society of Chemistry.

Onion‐like carbon and the hybrid of graphene nanosheet (GNS)CNT also illustrated the enhanced electrochemical performance to the LIB anode. Moreover, as the formation of solid electrolyte interphase in the first few cycles consumes a large amount of lithium ions from the cathode (LiCoO_2_, LiMn_0.5_Ni_0.5_O_2_, or LiFePO_4_) and exhibits low initial coulombic efficiency, the pre‐treatment of anode materials is an important approach to tackle this problem. Wang et al. synthesized an urchin‐like MoS_2_ composite using spherical onion‐like carbon core with a solvothermal and subsequent annealing treatment method. The new technique of pre‐lithiation of urchin‐like MoS_2_ composites by the direct contact with lithium foil is presented in **Figure** **11**a,b. During the intercalation process, the phase of MoS_2_ turn from 2H with semiconducting characteristics to 1T with metal features.^123^ The typical urchin‐like architecture of MoS_2_ composites is shown in Figure 11b,c. Compared to the commercial MoS_2_ powder, the urchin‐like MoS_2_ composite showed better electrochemical performance. The initial rechargeable capacity of urchin‐like MoS_2_ was 852 mA g^−1^ with a high Coulombic efficiency of 71.1% at the current density of 50 mA g^−1^ and the rate capability of urchin‐like MoS_2_ composite also demonstrated that the pre‐lithiation method can be applied to enhance the Coulombic efficiency for high‐performance LIBs (Figure 11d,e). The pre‐lithiated MoS_2_ nano‐urchins exhibited a high initial Coulombic efficiency of 97.6%, which was much higher than the normal anode electrode of LIBs. Although the initial capacity is slightly lower than that of normal MoS_2_ electrode, the highly stable cycling performance of pre‐lithiated MoS_2_ nano‐urchins paves the way to the commercialization of high‐performance LIBs. Additionally, our group successfully synthesized flower‐like MoS_2_‐GNS‐CNT composites.^124^ Because of the graphene backbone and the existence of CNTs, the further growth of MoS_2_ nanosheets was prevented (Figure 11e), which successfully restricted the thickness of MoS_2_ sheets in a narrow range of 5–10 nm (Figure 11f–h). This composite also has many advantages for application as a LIB anode because of the combination of the advantages of 0D MoS_2_ nanoparticles, 1D CNT, and 2D GNS. The CNTs performed the role of connecting graphene and MoS_2_ nanosheets and prevented the aggregation of adjacent graphene nanosheets. Graphene nanosheets were considered as the substrate for MoS_2_ nanosheets and also served as the fast electron‐conductive intermediate. Therefore, the nanocomposites exhibited a superior rate capability (the capacity of the nanocomposite was 830 mA g^−1^ at the current density of 0.5 A g^−1^) and long cyclability (a high capacity of 728 mA g^−1^ was remained at 5 A g^−1^ after 1000 cycles in Figure 11i,j), which is regarded as a promising candidate for LIB anode.^124^

### 2.2 Sodium Ion Batteries

Sodium ion batteries (SIBs) have attracted great attention in recent years as an alternative to the LIB rechargeable system because of the abundance of sodium resources, the even geographic distribution, and potentially low cost of the entire battery system. However, there are some disadvantages of SIBs that hinder their vast application. First, sodium ions in nature are almost three times heavier than lithium ions. Second, the radius of Na^+^ ions (1.06 Å) is much larger than that of Li^+^ ions (0.76 Å).^125^, ^126^, ^127^, ^128^, ^129^ Third, the low diffusion kinetics of Na^+^ ions, large volume expansion during sodiation, and pulverization of electrodes during cycling all result in the fast capacity decay and low Coulombic efficiency of SIBs. Therefore, many efforts have been devoted to address the problems of SIBs and develop appropriate cathode and anode materials. Graphite is usually applied as anode for commercial LIB, but it was found by Fouletier in 1988 that it is quite difficult to embed Na^+^ ions in the interlayers of crystalline graphite (the experimental capacity is only 36 mAh g^−1^). MoS_2_, with its exceptional physical and chemical features, is of great potential for application as an anode for SIBs.

**Figure 12:**
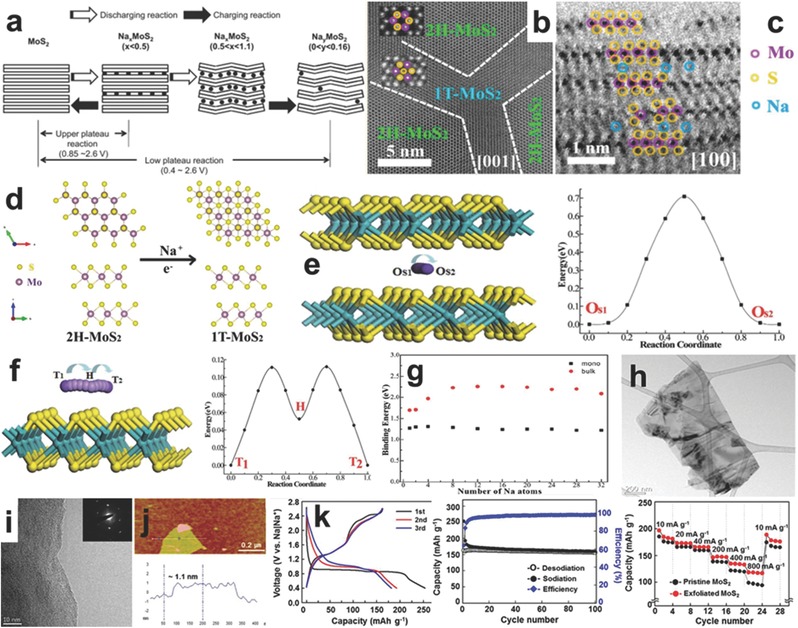
a) The scheme of the charge/discharge mechanism of Na/MoS_2_ half‐cells in the first cycle. Reproduced with permission.^130^ Copyright 2013, Elsevier.b,c) The high‐angle‐annular dark‐field (HAADF) image showing the transition of MoS_2_ phases and the Na^+^ ions intercalating in every other interlayer firstly. d) The scheme of the phase transition of MoS_2_ from 2H to 1T phase. e) The pathway of Na^+^ ion diffusion in the interlayer of bulk MoS_2_ and the corresponding energy profiles along the Na diffusion path. f) The Na migration path on the surface of monolayer MoS_2_ and the corresponding energy profile along the Na diffusion path. g) The relationship of binding energy and Na atoms in bulk MoS_2_ and monolayer MoS_2_. Reproduced with permission.^132^ Copyright 2014, Royal Society of Chemistry. h,i) TEM and HRTEM of MoS_2_ nanosheets and corresponding SAED pattern. j) The AFM and height profile of the MoS_2_ sheet. k) The charge/discharge profile, the cycling performance and rate capability of bulk MoS_2_ and exfoliated MoS_2_ nanosheets, respectively. Reproduced with permission.^133^ Copyright 2014, American Chemistry Society.

The better understanding of the phase transitions along with the lattice distortion and irreversible structural decomposition are critical to discover the code of the electrochemistry of MoS_2_ on SIBs, which sheds light on the material design and further industrial application. According the investigation of Ahn et al., the discharge/charge process of MoS_2_ in SIBs consists of two steps based on the inserted Na*_x_* amount (0 < *x* < 0.5, and 0.5 < *x* < 1.1). In the first stage, the crystal structure of MoS_2_ does not change due to the low inserted amount of Na ions. However, when the number of Na ions is in the range of 0.5 < *x* < 1.1, the layer structure of MoS_2_ continues expanding along the *c*‐axis, and causes slight structural distortion by inner strains, which is illustrated by the scheme in **Figure** **12**a. It is noted that the distorted structure of MoS_2_ cannot be fully recovered during charge due to the inner strains and irreversible decomposition during discharge.^130^ The structural transition of MoS_2_ during sodium intercalation was investigated on the atomic scale using in situ XRD and aberration‐corrected scanning transmission electron microscopy by Chen and co‐workers, as shown in Figure 12b,c. This demonstrates that the Na^+^ ions first insert in every other interlayer of the MoS_2_ nanocrystal, and then fully filled the other interlayer during discharge.^131^ Because of the large differences in the phase parameters of 2H‐MoS_2_, 2H‐Na_0.5_MoS_2_, 1T‐Na_0.5_MoS_2_, and 1T‐NaMoS_2_, as well as Na*_x_*S, the large strain and high energy barrier cause the phase transition from 2H‐ to 1T‐MoS_2_ (Figure 12d). The specific energy barriers for bulk MoS_2_ and graphene‐like monolayer MoS_2_ were studied by Wang et al.^132^ using the ab initio DFT method. The results demonstrated that the Na diffusion path in graphene‐like monolayer MoS_2_ was from two adjacent T‐sites to the nearest‐neighbor H‐site in a zigzag manner, and the active energy barrier for graphene‐like monolayer MoS_2_ is only 0.11 eV, which is much lower than bulk MoS_2_ (Figure 12e,f). Moreover, the Na binding energy of bulk MoS_2_ is gradually higher than that of monolayer MoS_2_ as the numbers of Na atoms increase (Figure 12g). The theoretical calculation suggests that graphene‐like MoS_2_ can show better cycling performance and high rate capability than bulk MoS_2_. After comparing the two types of MoS_2_ states, it is confirmed that layered MoS_2_ with the advantage of double‐sided adsorption can deliver a theoretical capacity of 335 mAh g^−1^ as an anode for SIBs. Based on this theory, many investigations have been applied to prepare monolayer MoS_2_ materials for SIBs. For example, Choi et al. prepared monolayer MoS_2_ using a facial liquid‐phase exfoliation in 1‐methyl‐2‐pyrrolidinone (PVP), demonstrating the typical few layer MoS_2_ nanosheets by TEM, HRTEM, and AFM (Figure 12h–j).^133^ When used as the anode of a SIB, the MoS_2_ nanosheets showed two plateaus at 0.94 V and 0.86 V and delivered a high discharge capacity of 254 mAh g^−1^. The MoS_2_ nanosheets exhibited good cycling performance in 100 cycles and rate capability (up to 800 mA g^−1^) compared to the bulk MoS_2_ materials (Figure 12k). and co‐workers designed two types of MoS_2_ nanomaterials (nanopetals and nanospheres).^134^, ^135^ When applied as anodes for SIBs, MoS_2_ nanopetals can maintain a capacity of 595 mAh g^−1^ at 0.1 C after 50 cycles with Na‐alginate as the binder, while the MoS_2_ nanospheres can only deliver a specific capacity of 520 mAh g^−1^ at 0.1 C. It is believed that exposed MoS_2_ edges mainly contribute to the well‐maintained capacity. A similar strategy to achieve monolayer MoS_2_ was used by Dou and Chou and co‐workers,^136^ who discovered that the exfoliated MoS_2_ composite with a carbon‐coated layer delivered a high capacity of 400 mAh g^−1^ at 0.25 C and maintained a long cycling life; most importantly, the carbon‐coated MoS_2_ nanosheets showed exceptional rate capability at 5 C (290 mAh g^−1^). Chen et al. prepared MoS_2_ nanoflowers with expended interlayers for SIB. By the control of cutting off voltage 0.4–3 V, the intercalation mechanism only took place, and the MoS_2_ nanoflowers delivered both high capacity and good cycling performance.^181^

**Figure 13:**
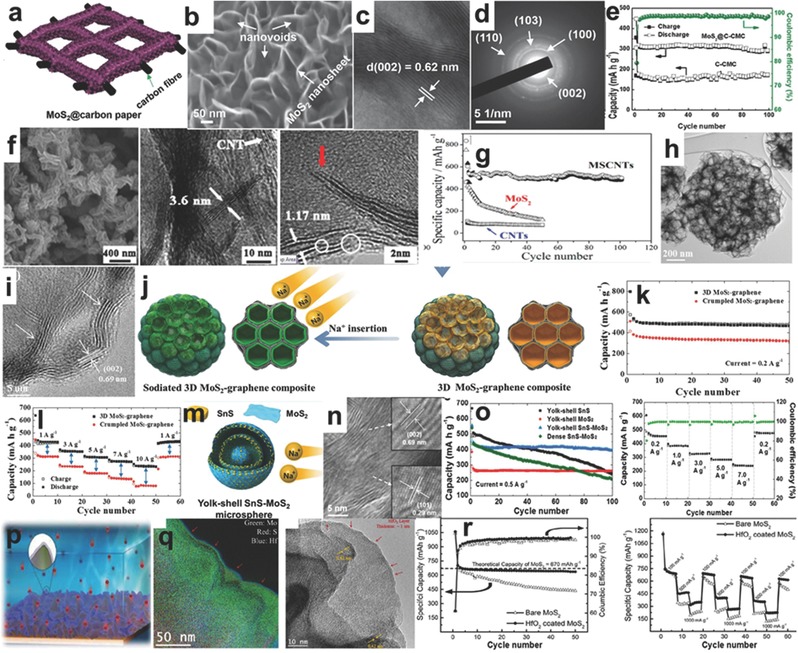
a) The scheme of growth MoS_2_ on carbon nanofibers (MoS_2_@C‐CMC). b–d) The SEM, TEM and SAED pattern of MoS_2_@C‐CMC. e) The cycling performances of MoS_2_@C‐CMC and C‐CMC, respectively. Reproduced with permission.^138^ f) The SEM, HRTEM images of MSCNTs composites. g) The comparison of cycling performances of MSCNTs, MoS_2_ and CNT, respectively. Reproduced with permission.^137^ Copyright 2014, American Chemical Society. h,i) The TEM and HRTEM images of 3D MoS_2_‐graphene composites. j) The scheme of the sodiation of 3D MoS_2_‐graphene composites. k,l) The cycling performance and rate capacity of 3D MoS_2_‐graphene composites. Reproduced with permission.^143^ m,n) The scheme and HRTEM image of yolk‐shell SnS‐MoS_2_ microsphere. o) The cycling performance and rate capability of yolk‐shell SnS‐MoS_2_ microsphere. Reproduced with permission.^144^ Copyright 2015, American Chemical Society. p,q) The scheme, TEM and elemental mapping images of HfO_2_ coated MoS_2_ nanosheets electrodes. r) The cycling performance and rate capacity of HfO_2_ coated MoS_2_ nanosheets. Reproduced with permission.^145^ Copyright 2015, Wiley‐VCH Verlag GmbH.

Although the graphene‐like monolayer MoS_2_ shows good electrochemical performance, the 2H/3R‐MoS_2_ with semiconductor properties should be enhanced in conductivity by a highly conductive intermediator, such as carbon nanofibers, carbon nanotubes, or graphene. The combination of MoS_2_ with graphene and carbon nanotubes can effectively enhance the electrochemical performance of SIBs, and the layered structure of MoS_2_ can be well‐maintained after long term cycles.^182^ For example, Zhang et al. prepared layered MoS_2_ grown on carbon nanotubes, which exhibited a specific capacity of 495.9 mAh g^−1^ at a current rate of 0.1 C and maintained 84.8% after 50 cycles. However, the 3D layered MoS_2_ nanosheets grown on graphene achieved a high capacity of 797 mAh g^−1^ at the same current rate (0.1 C) and about 99.98% of the initial capacity remained after 600 cycles, which demonstrates that the combination of layered graphene and MoS_2_ can maximize the advantages of the two materials and maintain a stable morphology after long term cycles.^137^ Xie et al. used the free‐standing carbon fibers derived from paper towel as the conductive substrate and loaded MoS_2_ nanosheets via hydrothermal method for binder‐free electrode without using a conductive agent.^138^ The scheme of the free‐standing MoS_2_@C is shown in **Figure** **13**a, demonstrating that interconnected carbon fiber can provide both a conductive mediator and a current collector. The SEM, TEM, and selected area electron diffraction (SAED) pattern in Figure 13b–d show the ultrathin MoS_2_ grown on carbon fiber, nanovoids in MoS_2_ nanosheets, and the typical phase of (002). The MoS_2_@C electrode with one‐step dip‐coating CMC approach formed MoS_2_@C‐CMC electrode. The MoS_2_@C‐CMC electrode delivered a high reversible capacity of 356 mAh g^−1^ at a current density of 80 mA g^−1^, which was much higher than the MoS_2_@C electrode. Moreover, the MoS_2_@C‐CMC electrode also exhibited superior cycling performance in Figure 13e. The good electrochemical performance of the MoS_2_@C‐CMC electrode is associated with the 3D interconnected carbon fiber for high conductivity and the ultrathin MoS_2_ nanosheets for fast Na^+^ ions diffusion. Zhu et al. synthesized interlayer‐expanded MoS_2_‐CNTs composite (MSCNTs) as an anode for SIBs using functionalized CNTs as backbones. The ultrathin MoS_2_ nanosheets with a space expanded structure were homogenously grown on the surface of CNTs (Figure 13f); the MoS_2_ nanosheets exhibit aninterlayer distance of 1.17 nm in the HRTEM image. The MSCNT electrode exhibited good cycling performance (delivering a reversible capacity of 504.6 mAh g^−1^ at 50 mA g^−1^, maintaining 495 mAh g^−1^ after 100 cycles in Figure 13g), which was much more stable than bare CNTs and the MoS_2_ electrode.^137^ In addition to CNTs and carbon nanofibers, graphene, a 2D layered structure with high conductivity and high flexibility,^63^, ^67^, ^139^, ^140^, ^141^ is another important substrate to support MoS_2_ nanosheets.^142^ With the assistance of the ultrasonic spray pyrolysis technique, Choi and co‐workers prepared MoS_2_ nanosheet‐coated carbon nanosphere composites in several tens of uniform nanospheres, and the size of nanospheres could be well controlled by the change of the PS nanobeads that served as template during the spray process as shown in Figure 13h–j.^143^ Compared to the crumpled MoS_2_‐graphene composite, the first discharge/charge capacities of 3D MoS_2_‐graphene spheres reached to 797 and 573 mAh g^−1^ at 0.2 A g^−1^, and maintained a high capacity of 480 mAh g^−1^, exhibited good rate capability (up to 10 A g^−1^), as shown in in Figure 13k,l. The outstanding electrochemical performance of 3D MoS_2_‐graphene spheres is ascribed to the 3D porous graphene microspheres offering enough inner voids for big volume changes, and an ultrathin layer of the MoS_2_ structure for fast Na^+^ ion diffusion.

Surface modification by metal sulfide and metal oxide on MoS_2_ is another approach to enhance the electrochemical performance of the MoS_2_ electrode. Kang et al. synthesized SnO_2_‐MoO_3_ yolk–shell microspheres by a one‐pot electrospray method and then successfully vulcanized to the SnS‐MoS_2_ yolk–shell microspheres as shown in Figure 13m. When used as an anode in SIBs, compared to dense‐structured SnS‐MoS_2,_ yolk–shell SnS and MoS_2_, yolk–shell SnS‐MoS_2_ microspheres exhibited a high capacity of 396 mAh g^−1^ after 100 cycles, while the capacity of dense‐structured SnS‐MoS_2_ only maintained 207 mAh g^−1^ in Figure 13o. The superior electrochemical performance is attributed to the synergetic effect of binary layered nanomaterials and the ultrastable yolk–shell structure.^144^ Surface passivation to layered MoS_2_ via an ultrathin and uniform HfO_2_ layer demonstrated by scheme, elemental mapping and TEM images in Figure 13p,q, another effective approach to maintain the performance of MoS_2_, was for the first time raised by Ahmed et al.,^145^ which largely improved the electrochemical performance of SIBs. The HfO_2_ coated MoS_2_ nanocomposites retained a high capacity of 636 mAh g^−1^ at the current density of 100 mA g^−1^, which was near the theoretical capacity of 670 mAh g^−1^, while the capacity of bare MoS_2_ only maintained 435 mAh g^−1^ at the 50^th^ cycle in Figure 13r. The exceptional results were possibly attributed to the ultrathin and uniform HfO_2_ layer, which acted as the passivation layer and prevented the structural degradation of MoS_2_ layers. Meanwhile, the amorphous features of HfO_2_ allowed for the facile diffusion of Na^+^ ions during the cycles. Therefore, the understanding of the phase transformation, surface modification, and morphology design of MoS_2_ are of significant importance to enhance the electrochemical performance and shine light for the practical application of SIBs.

### 2.3 Supercapacitors

Supercapacitors, divided into double‐layer capacitors, pseudocapacitors, and hybrid capacitors by the energy storage mechanisms, are a new‐type of electrochemical capacitor with high capacitance. They bridge the gap between electrolytic capacitors and high‐energy‐density secondary batteries. Compared to traditional capacitors, supercapacitors have the characteristics of high energy density, high cycling life span, short charging time, high rate of power, and strong resistance to the environmental temperature. These advantages allow them to be applied widely on the portable electronic devices and the state of vehicles.^146^ The electric double‐layer capacitors normally use carbon‐based materials with ultrahigh high surface areas as electrodes, forming the separation of charge at the interface between the two electrodes and the electrolyte. Pseudocapacitors usually employ metal oxide or conducting polymers as the electrodes, which work by quick and reversible Faradaic electron charge‐transfer with redox reactions, intercalation, or electrosorption in the active materials.^147^ Hybrid capacitors contain electrodes with different features: one side shows electrostatic capacitance and the other side exhibits the electrochemical capacitance, such as a lithium‐ion capacitor. Various carbon‐based materials and metal oxides with high surface area have been reported for high performance supercapacitors. MoS_2_, a 2D layered material with ultrahigh surface area and mechanical properties, is regarded as one of the most promising candidates for supercapacitors. **Table** **1** shows a brief comparison of recent various investigations on the supercapacitor applications of MoS_2_. Fan and co‐workers successfully prepared the flower‐like MoS_2_/C nanospheres using a facile hydrothermal method. The specific capacitance of flower‐like MoS_2_/C nanospheres reached 201.4 F g^−1^ at a current density of 0.2 A g^−1^, demonstrating that layered MoS_2_ has the natural advantage on the application of supercapacitor.^148^

**Table 1:** The comparison of MoS_2_ composites with different morphologies for the electrodes of supercapacitors

Topography	Component	Capacity	Life span	Ref.
Flower‐like nanospheres	MoS_2_ and C	201.4 Fg^‐1^	keeps 89.4% of the initial capacitance at a current density of 0.2 A g^‐1^	148
Nanosheets	2D metallic 1 T phase MoS_2_ sheets and various ions	From 70 to 400 F cm^‐3^	Remain 95% after 1000 cycles	149
Nanosheets	Pure MoS_2_	Can keep 330 F cm^‐3^ at a current density of 25.47 mA cm^‐2^	About 97% capacitance can be kept over 5000 cycles	40
Nanosheets	MoS_2_ and graphene	The capacitances measured values at 10 mV s^‐1^ are 128, 265, and 148 F g^‐1^ of the MoS_2_/rGO nanocomposites in the low, medium, and high concentrations.	Nearly 92% of the initial specific capacitance retained after 1000 cycles	151
Nanosheets	PANI/MoS_2_	575 F g^‐1^ at 1 A g^‐1^.	Less than 2% decrease in the capacitance after 500 cycles at the current density of 1 A g^‐1^	152
3D nanocomposite	MoS_2_/MWCNT	Reach 452.7 F g^‐1^ at a current density of 1 Ag^‐1^	After 1000 cycles, the fade was about 4.2%	153
Flower‐like 3D composite	MoS_2_‐carbon aerogel	260.0 F g^‐1^ at 1A g^‐1^	More than 92% can be retained after 1500 cycles	154
Hierarchical nanosphere	MoS_2_	368 F g^‐1^ at a scan rate of 5 mV s^‐1^	Retain 96.5% after 5000 cycles at a current density of 0.8 mA	155
Nanobar	C@Ni_3_S_2_@MoS_2_	Can reach to nearly1544 F g^‐1^ when the current density was 2 A g^‐1^	After 2000 cycles, the capacity can be remained about 92.8% when the current density was 20 A g^‐1^	156
Nanofibers	Ti/TiO_2_/MoS_2_	230.2 Fg^‐1^ (70.6 F cm^‐3^)	Show an energy density of 2.70 Wh kg^‐1^ (4.98 mWh cm^‐3^) while power density was 530.9 W kg^‐1^ (977.4 mW cm^‐1^)	157
Nanospheres	MoS_2_ modified with the assistance of sodium alginate	145 F g^‐1^ at the current density of 3 A g^‐1^	Null	30
MoS_2_‐graphene hybrid films	MoS_2_ and graphene	282 F g^‐1^ at a scan rate of 20 mV s^‐1^	Maintain 93% of its initial capacitance over 1000 cycles	158
Nanosheets	1H MoS_2_@ olwylamin	The capacity can reach to 50 mF cm^‐2^	Null	159
Core‐shell structure	Ni_3_S_4_@MoS_2_	1440.9 Fg^‐1^ at 2 Ag^‐1^	90.7% can be remained after 3000 cycles	160

**Figure 14:**
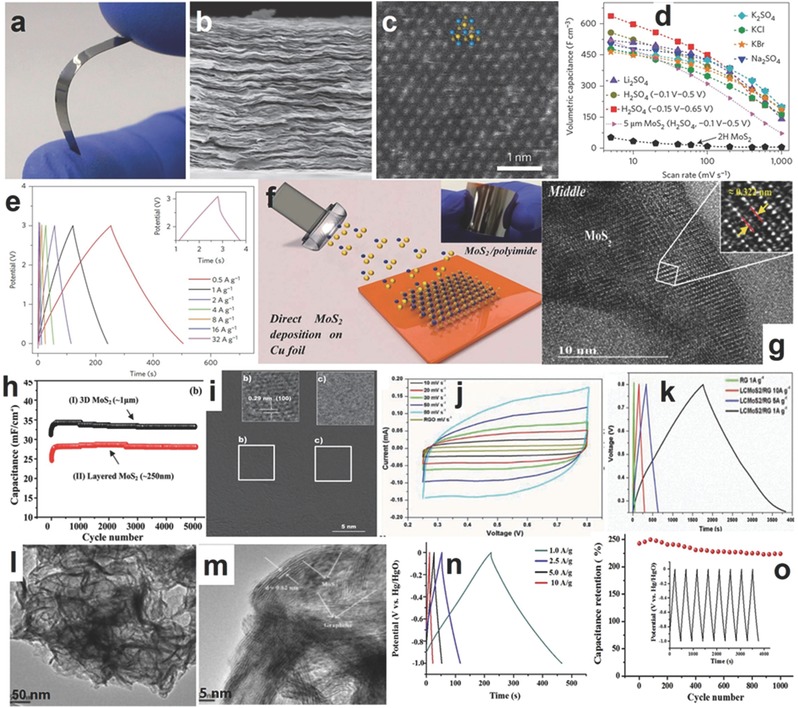
a) The digital photograph of MoS_2_ film. b,c) The SEM image and HRTEM image of the layered MoS_2_, showing the 1T phase of MoS_2_. d,e) The comparison of volumertric capacitance of 1T phase MoS_2_ electrode in different electrolytes and the charge/discharge profiles. Reproduced with permission.^167^ Copyright 2015, Nature Publishing Group. f,g) The mechanism of the direct magnetron sputtering technique and HRTEM observation of the 3D MoS_2_ film, respectively. Reproduced with permission.^158^ Copyright 2016, Royal Society of Chemistry. h) The comparisons of 3D MoS_2_ film and other electrode materials. Reproduced with permission.^150^ Copyright 2016, Royal Society of Chemistry. i) The SEM image, AFM image and profiles of LCMoS_2_@RGO. j,k) The CV curves and charge/discharge profiles of LCMoS_2_@RGO.^161^ l,m) The TEM and HRTEM image of MoS_2_–G composites. n,o) The charge/discharge profile, rate capacitance and cycling performance of MoS_2_‐Gr composites. Reproduced with permission.^163^ Copyright 2013, Elsevier B. V.

Chemical exfoliation is an economic approach to achieve large‐scale and high‐quality MoS_2_ nanosheets, and the chemically exfoliated MoS_2_ nanosheets exhibit outstanding electrochemical performance as the electrode in supercapacitors. For example, Acerce et al. chemically exfoliated MoS_2_ nanosheets and prepared flexible and free‐standing MoS_2_ films containing a high concentration of the metallic 1T phase as shown in **Figure** **14**a–c, which can electrochemically intercalate with different ions (H^+^, Li^+^, Na^+^, and K^+^) with a high capacitances ranging from 400 to 700 F cm^−3^ in different types of aqueous electrolytes (Figure 14d). The layered MoS_2_ film was also demonstrated to be suitably applied under the high‐voltage (3.5 V) operation in supercapacitors with organic electrolytes, and exhibited high volumetric energy and power density values as well as a high Coulombic efficiency of 95% over 5000 cycles (Figure 14e). The superior electrochemical performances are ascribed to the favorable electrochemical features (high hydrophilicity and high electrical conductivity) of 1T MoS_2_ layers analyzed using XRD, which can dynamically expand and facilitate the intercalations of various ions.^149^ Choudhary and co‐workers explored large‐scale and thickness‐modulated MoS_2_ sheets using a two‐step sputtering–CVD method that showed the wafer‐scale fabrication and thickness modulation of MoS_2_ layers from monolayer to multilayers (Figure 14f,g). The as‐prepared MoS_2_ sheets with much higher field‐effect mobility and current on/off ratio were applied as field‐effect transistors and revealed a p‐type semiconductor behavior that was much higher than that of an amorphous silicon (a‐Si) thin‐film supercapacitor (Figure 14h).^150^

To investigate the influence of the synergetic effect between MoS_2_ and graphene nanosheets, the combination of RGO sheets with MoS_2_ sheets was realized by the microwave‐heating technique, which supply energy to facilitate the formation of covalent chemical bonds of RGO and MoS_2_. Leite et al. synthesized the MoS_2_/RGO nanocomposites with different concentrations via microwave radiation technique as shown in Figure 14i (the SEM and HRTEM images of low concentration of MoS_2_/RGO nanocomposites are shown).^161^ It was demonstrated that the first layers of MoS_2_ are directly bonded with RGO via the aforementioned covalent chemical bonds, which were reversibly measured between 0.25 and 0.8 V in 1 m HClO_4_ for low concentrations of MoS_2_ and 0.25 V–0.65 V for medium and high concentration of MoS_2_ layers on graphene (Figure 14j,k). The current density of low concentration MoS_2_/RGO hybrid was 63 Wh kg^−1^, and the supercapacitor electrode exhibited exceptional cycling stability (92% capacitance remained after 1000 cycles), which illustrated that the supercapacitance can be altered via the changes of MoS_2_ concentrations in hybrids. Similarly, Byun and co‐workers synthesized a MoS_2_/GO hybrid film by the addition of GO sheets, which performed as the phase stabilizer and mechanical supporter. The MoS_2_/GO hybrid film was treated by a post‐annealing process in air to form the high concentration of distorted 1T‐ MoS_2_ for the high conductivity. Therefore, the MoS_2_/GO hybrid film as supercapacitor electrodes exhibited enhanced electrochemical performance, such as high volumetric capacitance (≈380 F cm^−3^ at the scan rate of 10 mV s^−1^), ultrahigh volumetric power density (≈207 W cm^−3^), and a good cycling life span (95% retention after 10 000 cycles) in the condition of the aqueous electrolyte.^162^ Huang et al. made a comparison of the electrochemical performances of three materials (pure graphene, pure MoS_2_, and MoS_2_‐graphene composites) as shown in Figure 14l,m.^163^ When applied as a supercapacitor anode, the specific capacitance was 243 F g^−1^ at a current density of 1 A g^−1^. The energy density was up to 73.5 Wh kg^−1^ when the powder density was 19.5 kW kg^−1^ (Figure 14n). Good cycling performance of MoS_2_‐graphene composites was also achieved for 1000 cycles, as shown in Figure 14o. That is attributed to the interconnected and conductive network of 3D MoS_2_‐graphene composites, which facilitated the fast charge transport and electrolyte diffusion and also buffered the volume expansion/contraction during cycles. Nitrogen‐doped graphene is also an attractive material for improving the performance of the MoS_2_ nanosheets and has been widely investigated for supercapacitors. The capacity could reach 245 F g^−1^ when the current was 0.25 A g^−1^ and after 1000 cycles, the retention was 91.6% which was very ideal.^164^ Graphene aerogel composites are also a common material for modifying the performance, and a kind of 3D mesoporous MoS_2_ composite can be made. This material promises the stable distribution of 2D nanosheets and the ideal effective electron and ion transfer. The capacity can reach 268 F g^−1^ when the current was 15 mA g^−1^ and a good cycle life span with about 93% remained after 1000 cycles. The ratio of Mo and S was 31.8% and 68.2% atomic. The BET surface area was about 149.3 m^2^ g^−1^ with the proven volume of about 0.21 cm^3^ g^−1^.^165^ By the way, the sandwich‐like MoS_2_ and graphene composite material also has unique structure characteristics as indicated by its perfect electrochemical performance. Most of the MoS_2_ samples are amorphous, which is hard to maximize the performance of MoS_2_. To synthesize highly crystalline MoS_2_ atomic layers, Patil and co‐workers used the facial exfoliation method to prepare crystalline MoS_2_‐graphene sheets in a layer‐by‐layer structure.^158^ The MoS_2_‐graphene hybrid film delivered a specific capacitance of 282 F g^−1^ when the scan rate was 20 mV s^−1^. It also showed much improved cycle life with a capacitance fade rate of 7% after 1000 cycles.^158^ Moreover, the chemically exfoliated MoS_2_ with high surface area and good quality can be well maintained with the combination of graphene sheets. Bisset et al. prepared a flexible MoS_2_‐graphene membrane as the electrode of a supercapacitor using an aqueous electrolyte, and it was found that the specific capacitance could be altered by reducing the resistivity of the electrodes and the morphology of the membrane. During the long‐term cycling, it was demonstrated that the capacitance could be increased (up to 800%) due to the partial re‐exfoliation of the layered materials via ion intercalation.^166^

**Figure 15:**
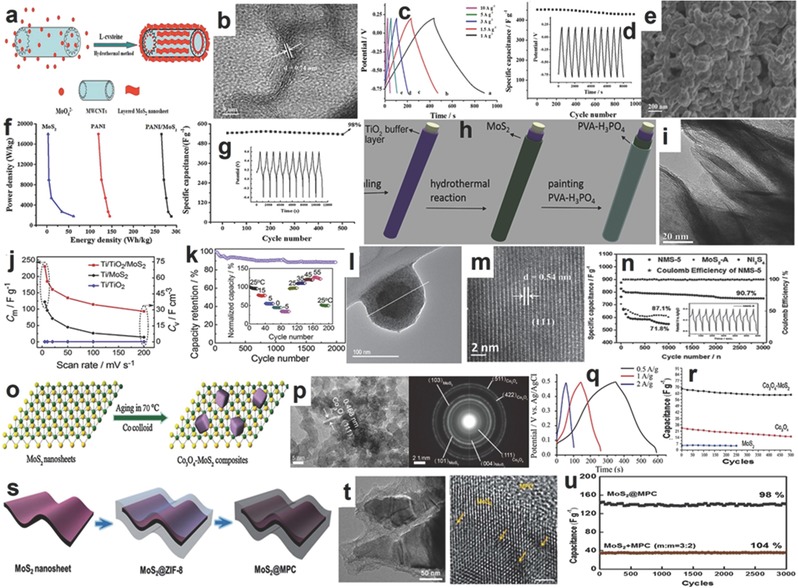
a) The scheme of preparing MoS_2_/MWCNT composites. b) The HRTEM image of MoS_2_/MWCNT composites. c,d) The charge/discharge profile and cycling performance of MoS_2_/MWCNT composites. Reproduced with permission.^169^ Copyright 2014, Elsevier. e) The SEM image of PANI@MoS_2_ composites. f,g) The rate capacity and cycling performance of PANI@MoS_2_ composites. Reproduced with permission.^152^ Copyright 2013, Elsevier. h‐i) The scheme and HRTEM image of TiO_2_@MoS_2_ composites. j,k) The capacitance and cycling performance of TiO_2_@MoS_2_ composites. Reproduced with permission.^171^ Copyright 2014, Elsevier. l,m) The TEM, elemental distribution profile and HRTEM of HRTEM images of Ni_3_S_4_@amorphous MoS_2_ composite. n) The cycling performance of Ni_3_S_4_@amorphous MoS_2_ composite. Reproduced with permission.^160^ o,p) The scheme of Co_3_O_4_@MoS_2_ preparation, and the TEM and SAED pattern of Co_3_O_4_@MoS_2_ composite. q,r) The charge/discharge profile and cycling performance of Co_3_O_4_@MoS_2_ composite Reproduced with permission.^174^ Copyright 2015, American Chemistry Society. s) The scheme of MoS_2_@MPC preparation. t) The SEM, TEM and HRTEM images of MoS_2_@MPC. u) The cycling performance of MoS_2_@MPC. Reproduced with permission.^177^ Copyright 2015, Royal Society of Chemistry.

Additionally, tubular C/MoS_2_ nanocomposites were synthesized by Hu et al. using porous anodic aluminum oxide as the template. When applied as supercapacitor electrode, the tubular C/MoS_2_ nanocomposites exhibited a high capacitance of 210 F g^−1^ at a current density of 1A g^−1^ and superior cycling performance (over 1000 cycles).^168^ By combining the multiwalled carbon nanotube and MoS_2_ with the 2D graphene‐like structure, the MoS_2_/MWCNT nanocomposites were successfully prepared as shown in **Figure** **15**a,b,^169^ when examined as supercapacitor electrode, the MoS_2_/MWCNT nanocomposites exhibited a specific capacitance of 452.7 F g^−1^ at a current density of 1 A g^−1^, which was much higher than that of bare MWCNTs (69.2 F g^−1^) and bare MoS_2_ (149.6 F g^−1^). Only about 4.2% of the capacitance was observed after 1000 cycles at a current density of 1A g^−1^ in Figure 15c,d, demonstrating the apparent synergetic effect among graphene, MCNT and MoS_2_. The flower‐like MoS_2_ incorporated by carbon aerogel was synthesized by a facile hydrothermal route assisted by l‐cysteine. The MoS_2_/carbon aerogel hybrid film delivered a high capacitance of 260.0 F g^−1^ at 1 A g^−1^ in the condition of 1.0 m Na_2_SO_4_ aqueous solution,and exhibited a stable cycling performance (92.4% of the initial capacitance was retained after 1500 cycles).^154^

In addition to the carbon based materials for modifying the features of MoS_2_, conductive polymers are also used to modify the MoS_2_ sheets. Huang et al. prepared polyaniline/molybdenum disulfide (PANI/MoS_2_) using a facile in situ polymerization method, which provided direct paths for electrons via PANI (Figure 15e).^152^ The layered structure of PANI/MoS_2_ facilitated the contact surface area, which was in favor of the intercalation/deintercalation of protons during cycles. The PANI/MoS_2_ nanocomposites delivered a high capacitance of 575 F g^−1^ at a current density of 1 A g^−1^ (Figure 15f). Additionally, the PANI/MoS_2_ exhibited a superior cycling performance (≈2% capacitance decrease was observed after 500 cycles at 1 A g^−1^) in Figure 15g, which was ascribed to the synergistic effect of PANI and MoS_2_. The combination of PANI and MoS_2_ was also demonstrated by Liu et al. via the 3D tubular structure with the MoS_2_ nanotube as core and PANI nanowire arrays as shell that grew on both sides of MoS_2_ nanotubes. When the amount of the PANI loading on the MoS_2_ was 60%, the MoS_2_/PANI‐60 hybrid electrode exhibited a high capacitance of 552 F g^−1^ at a current density of 0.5 A g^−1^, which also showed exceptional rate capability of 82% from 0.5 to 30 A g^−1^. The MoS_2_/PANI‐60‐based symmetric supercapacitor illustrated good rate capability and cycling stability. Specifically, the capacitance based on the two electrodes was 124 F g^−1^ at the current density of 1 A g^−1^, and around 79% of the initial capacitance was well maintained after 6000 cycles, which demonstrated the stable tubular structure and the synergetic effects of PANI and MoS_2_ hybrid.^170^

The coating of transition metal/metal oxides or metal sulfides on MoS_2_ is in favor of the structural stability of MoS_2_ and improves the electrochemical performance of supercapacitor electrodes. For example, the Ti/TiO_2_/MoS_2_ coaxial fibers with a fabricated TiO_2_ buffer layer and MoS_2_ nanosheets grown on Ti nano­wires via a hydrothermal method exhibited flexible features and strong mechanical properties (Figure 15h,i). When fabricated for the symmetrical supercapacitor, the capacitance of Ti/TiO_2_/MoS_2_ fibers reached 230.2 F g^−1^ with an energy density of 2.70 Wh kg^−1^ (Figure 15j,k). Moreover, the Ti/TiO_2_/MoS_2_ fibers also exhibited exceptional process abilities on stretchable, spring‐shaped devices to light LEDs.^171^ Kim and co‐workers prepared MoS_2_ sheets on the Mo foil using a hydrothermal method and demonstrated the binder‐free electrode for supercapacitors. The cyclic voltammetric figure illustrated the combination of pseudocapacitor and electrochemical double layer capacitance for the MoS_2_‐Mo foil. That caused it to deliver a capacitance of 192.7 F g^−1^ when the current density was 1 mA cm^−2^. It was possibly ascribed to the semiconducting nature of 2H‐phase MoS_2_.^172^ Li et al. have synthesized hierarchical carbon‐coated Ni_3_S_2_@MoS_2_ double core–shell nanorods via a facial hydrothermal method with the carbon‐coated nickel nanorods as the precursor and template. When used as a supercapacitor electrode, the C@Ni_3_S_2_@MoS_2_ nanorods exhibited a high capacitance of 1544 F g^−1^ when the current density was 2 A g^−1^ and had a good life span (the capacitance remained 92.8% at the current density of 20 A g^−1^ after 2000 cycles). The good electrochemical performance was attributed to the backbone of C/Ni nanorods on increasing rate performances and the interconnected MoS_2_ nanosheets on providing numerous accessible surfaces and the good contact with the electrolyte.^156^ Another good example of the combination of metal sulfide (Ni_3_S_4_) with MoS_2_ nanospheres is illustrated by Zhang et al. (Figure 15l,m). They first confirmed that amorphous MoS_2_ showed 1.6 times the capacitance of the crystalline one.^160^ Then, they applied the prepared crystalline core@amorphous shell (Ni_3_S_4_@MoS_2_) by a facile one‐pot process for the supercapacitor. The Ni_3_S_4_@amorphous MoS_2_ nanospheres exhibited a high capacitance of 1440.9 F g^−1^ when the current density was 2 A g^−1^ and exceptional cycling performance (90.7% remained capacitance was achieved after 3000 cycles at 10 A g^−1^) in Figure 15n, which is ascribed to the advantages of the flexible protection of the amorphous shell and a highly conductive core. The excellent performance made it a good candidate for the electrode of supercapacitors.^160^ Moreover, another type of Ni_3_S_2_@MoS_2_ composite was synthesized by Wang et al. via a green and scalable one‐step method.^173^ The heterostructure of the Ni_3_S_2_@MoS_2_ composite delivered a capacitance of 848 F g^−1^, which was almost twice that of pure Ni_3_S_2_ (425 F g^−1^). After 2000 cycles, the capacitance retention of Ni_3_S_2_@MoS_2_ composite was about 97%. This was attributed to the core–shell structure and the high surface area of Ni_3_S_2_@MoS_2_ composite. The metallic oxide nanoparticles endow MoS_2_ nanosheets with different properties. For example, Wang and co‐workers fabricated MoS_2_‐Co_3_O_4_ composites using laser ablation in liquids and an aging‐induced phase transformation method (Figure 15o). They used reactive Co colloids that acted as none ion precursor to anchor Co_3_O_4_ nanoparticles on the surface of MoS_2_ nanosheets without the bridging agent, thereby the phenomenon that the existence of Co_3_O_4_ can be strengthened the stability of the MoS_2_ layers can be confirmed (Figure 15p). The MoS_2_‐Co_3_O_4_ composites exhibited a specific capacity of 69 mAh g^−1^ at a current density of 0.5 Ag^−1^ and maintained 87% capacitance after 500 cycles (Figure 15q,r), which was higher than that of bare MoS_2_ or Co_3_O_4_.^174^ Wang et al. used MnCO_3_ nanospheres as a template to synthesize MoS_2_ hierarchical hollow nanospheres.^175^ The capacitance was 142 F g^−1^ when the current was 0.59 A g^−1^ and could keep 92.9% after 1000 cycles. The flower‐like structure has been investigated for a long time. The MoS_2_ that was synthesized by Wang achieves the neat structure. The capacity was 168 F g^−1^ when the current was 1 A g^−1^ and approximately 92.6% was retained after 6000 cycles.^176^

Hybrid supercapacitors with a different electrochemical mechanism with one side of electrostatic attractions and another side of electrochemical reactioncan maximize the energy storage and also maintain the power density. Wang et al. proposed a proof‐of‐concept of a hybrid supercapacitor with a pseudocapacitive core of MoS_2_ nanosheets and an electrostatic double layer capacitive shell of microporous carbons (MPC), which is converted from MOF (zeolitic imidazolate framework: ZIF‐8; Figure 15s).^177^ Mesoporous carbon was homogenously coated the surface of MoS2 nanosheets and showed 1T‐phase structure (Figure 15t). The as‐prepared MoS_2_‐MPC nanosheets exhibited a capacitance of 189 F g^−1^ when the current was 1 A g^−1^ as shown in Figure 15u. It kept 98% of capacitance after 6000 cycles, which is attributed to the combination of structural stability and electrochemical charge storage.

## 3 Conclusion and Outlook

In summary, we have introduced the unique properties of mono­layer MoS_2_ and special characteristics of MoS_2_ composites after various post‐treatment and surface modifications and reviewed recent progress in the applications of LIBs, SIBs, and supercapacitors. The main focus is on the material preparation, structural characterization, energy storage applications, and possible intercalation mechanisms that occurred in the electrochemical reactions. Limitations for MoS_2_ are identified and numerous approaches are introduced. We hope to inspire more creative ideas and novel techniques to maximize electrochemical performance of MoS_2_ and shorten the distance between laboratory research and commercial applications. To achieve that, theoretical calculations and experimental works all demonstrated that interfacial modifications and structural controls could significantly enhance the specific capacity, rate performance, and cycling life span of MoS_2_ nanocomposites. Phase structure tuning and changes of the crystalline purity are also potential tools for enhancing the electrical transport properties of MoS_2_. With the increasing amount of fundamental research on MoS_2_, the challenges of MoS_2_ with drawbacks and unstable feature of will be addressed, and the vast applications for energy storage and electrochemical catalysis of MoS_2_ and similar metal sulfides/selenides (WS_2_, MoSe_2_ and WSe_2_) will be possible in the near future.
